# Energetic Controls on Biodegradation of Organic Matter from Diverse Environmental Sources

**DOI:** 10.1021/acs.est.5c18534

**Published:** 2026-09-10

**Authors:** Konstantinos-Marios Vaziourakis, Liam Heffernan, Elizabeth Jakobsson, Dolly Kothawala, Lars Tranvik

**Affiliations:** † Department of Ecology and Genetics/Limnology, 174436 Uppsala University, Norbyvägen 18 D, Uppsala 752 36, Sweden; ‡ Department of Earth Sciences, Vrije Universiteit Amsterdam, De Boelelaan 1085, Amsterdam 1081 HV, The Netherlands

**Keywords:** organic matter, degradation, energy content, Gibbs free energy, microbial metabolism, respiratory quotient, ecosystems

## Abstract

Organic matter (OM) decomposition is a central process in the global carbon cycle, regulating whether OM accumulates as a long-term sink or is mineralized into greenhouse gases. Although multiple explanations have been proposed to what controls OM degradation, the energetic and thermodynamic properties of OM have received less attention, particularly in cross-system studies. We incubated a wide range of OM from soils, sediments, and plant material, simultaneously measuring CO_2_ production and O_2_ consumption to quantify decomposition rates and the respiratory quotient (RQ, i.e., CO_2_/O_2_ molar ratio) of the degrading communities. We also analyzed various OM properties, including energy content and intrinsic thermodynamic stability (*ΔG*_*f*_, Gibbs free energy of OM formation), to assess how these factors explain the variability in decomposition rates and the metabolic degrading capacity across ecosystems. Our results showed that *ΔG*_*f*_ and specific energy content (both expressed in kJ g C^–1^) can substantially influence the degradation rates across environmental samples, and that OM–mineral interactions can further constrain decomposition. The RQ ranged from 0.34 in low-organic deposits to 1.36 in fresh plant material, showing similar trends to degradation rates and indicating differences in substrate use and metabolic capacity. This is the first cross-system study linking microbial degradation to the energetic signature of OM, revealing how intrinsic energetic properties can explain the variability in carbon persistence across a wide range of natural OM sources.

## Graphical abstract

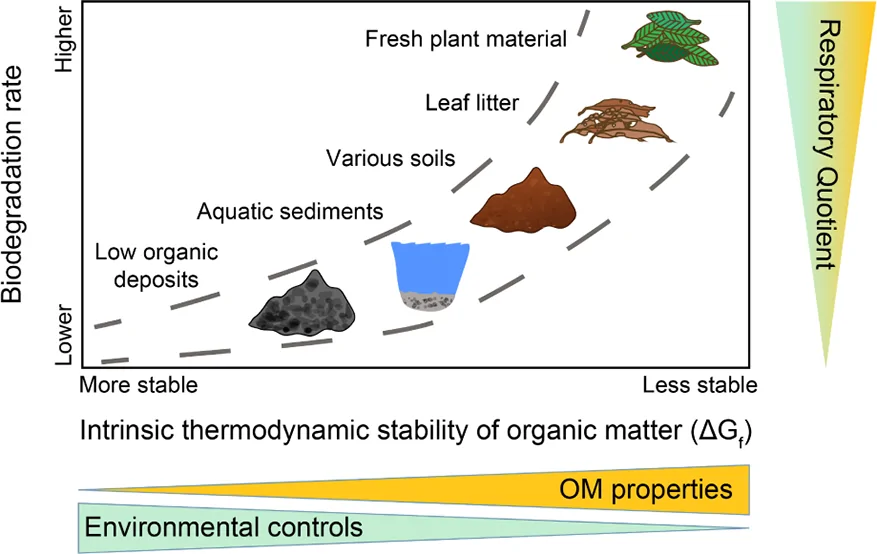

## Introduction

Organic matter (OM) is a key component of Earth’s ecosystems, regulating biogeochemical processes and elemental cycling.^1^ Microbial decomposition determines whether OM persists as a long-term sink or is mineralized into greenhouse gases;^2^ thus, understanding the factors that control the rate of microbially derived OM decay is crucial. Several factors have been proposed to control biodegradability in different ecosystems; the soil literature increasingly emphasizes extrinsic environmental controls such as soil heterogeneity and mineral adsorption,^3,4^ whereas freshwater studies highlight intrinsic molecular properties as major constraints on OM degradation.^5–7^ These divergent perspectives suggest that the understanding of broader mechanistic controls spanning distinct environmental boundaries is poorly developed.^8^

Decomposition is commonly considered to depend on the molecular composition of OM, including bulk proxies such as the carbon-to-nitrogen ratio (C:N),^9–11^ or structural and molecular characterization based on Fourier-transform infrared spectroscopy (FTIR)^12,13^ and solid-state nuclear magnetic resonance spectroscopy (NMR).^14–16^ In addition to the solid phase, the properties of dissolved organic matter (DOM) can also be analyzed for its persistence in soils^17^ and aquatic environments^5^ using bulk optical characteristics (absorbance and fluorescence) of DOM^6,18,19^ and high-resolution mass spectrometry^20,21^ to describe molecular characteristics. Although all of these methods are helpful for understanding OM chemical composition, they are not straightforward in assessing how energetically and thermodynamically accessible OM is to microbial decomposition. To bridge this gap, it is essential to evaluate the extent to which the energetic and thermodynamic traits of OM are linked to its degradability.

While bioenergetic frameworks have been proposed for soil ecosystems,^22–30^ empirical studies that apply them across diverse environmental samples remain scarce. The specific energy, i.e., the bulk, calorimetrically measured energy per unit organic carbon, reflects the energy stored in the chemical bonds of organic substrates and has been shown to decrease with decomposition, with more persistent OM in soils being less energy dense.^24,28,29,31^ This supports the idea that the energy content of the OM reflects the fraction of OM that is available to microbial degraders. Accordingly, microbial degraders preferentially utilize high-energy OM, while the degradation of lower-energy substrates may involve greater enzymatic investment and yield a lower net energy. Thus, the microbial processing of OM in soils and sediments under aerobic conditions, where the variability due to alternative electron acceptors is minimized, is mechanistically favored by substrates that provide higher available energy to the microbial decomposers.^29,31–36^

From a thermodynamic perspective, the intrinsic stability of OM, expressed as its Gibbs free energy of formation (*ΔG*_*f*_), indicates its energetic favorability for microbial oxidation. *ΔG*_*f*_ differs from the Gibbs energy of a decomposition reaction, which depends on reaction stoichiometry and the terminal electron acceptor and is difficult to constrain for heterogeneous OM mixtures.^27^ Instead, *ΔG*_*f*_ integrates the stoichiometric and energetic properties of the substrate to characterize its intrinsic thermodynamic stability, independent of specific reaction pathways or electron acceptor availability.^30,37^ OM with higher *ΔG*_*f*_ is less thermodynamically stable and thus more favorable for microbial degradation, whereas lower *ΔG*_*f*_ indicates greater stability and higher energetic requirements for oxidation under aerobic conditions, where oxygen is the dominant electron acceptor. *ΔG*_*f*_ can be calculated using bomb calorimetry and elemental analysis^30,38^ and as a concept, has mainly been used in studies of peat to interpret carbon accumulation,^30,37^ while its broader application across natural OM sources is still limited. While *ΔG*_*f*_ reflects intrinsic thermodynamic stability, actual decomposition rates can be influenced by kinetic environmental constraints, as reflected by the activation energy (*Ea*).^8,27,29^ As a result, thermodynamically favorable OM may persist under conditions where environmental limitations restrict degradation. Yet, it remains unclear whether intrinsic energetic properties can predict OM biodegradability beyond ecosystem-specific kinetic constraints.

In addition to the energetic constraints of bioavailable OM, the degradative capacity of microbial communities, including their physiological state and capacity to produce the required extracellular enzymes, also influences decomposition, as reflected in their respiratory quotient (RQ), which is primarily regulated by growth stoichiometry.^39–41^ The RQ is the molar ratio of carbon dioxide produced to that of oxygen consumed during aerobic respiration. On the basis of the intrinsic properties of OM, highly oxidized substrates tend to result in higher RQ values, whereas more reduced substrates are associated with lower RQ values.^40,42^ Recently, it was suggested that the metabolic capacity of microbial degraders to utilize various carbon substrates controls RQ, and that the intrinsic properties of OM did not substantially affect variation in RQ.^41^ However, it remains poorly understood whether variability in RQ captures cross-system patterns in decomposition rates and is influenced by the energetic and thermodynamic properties of OM.

Studies of the mineralization of OM rarely compare across broad ranges of environmental sources. It has been argued that wide cross-system comparisons could provide a unified understanding of OM degradation.^8^ Based on this argument, we incubated OM from litter, soil, and sediment from various aquatic and terrestrial environments to explore their aerobic decomposition dynamics. We aim to (a) identify key characteristics of OM, particularly those related to its energy availability (kJ g C^–1^), controlling its microbial degradability, and (b) test whether a simple model can predict OM degradation rates across a wide range of soils and sediments with different characteristics across different climatic regions. We hypothesized that, despite the vast diagenetic differences and the ambient microbiome of fresh plant material, leaf litter, soils, and aquatic sediments, the energetic properties (specific energy and *ΔG*_*f*_) of OM, relative to other OM descriptors, can control its biodegradability. Therefore, both energetic and thermodynamic traits of OM can represent a universal link between the intrinsic properties of OM and microbial degradation.

## Materials and Methods

### Sample Collection and Preparation

We collected OM from litter, soil, and sediment across 41 sites between September 2023 and March 2024. Samples were retrieved from 8 different environmental sources, i.e., topsoil (O and A horizon) under forest vegetation, named hereafter as forest soils (*n* = 5); topsoil (O and A horizon) of agriculturally managed sites, named hereafter as agricultural soils (*n* = 6); subsoils and parent material (B and C horizon), named hereafter as low organic deposits (*n* = 3); organic soils, including histosol from peatlands and histel from permafrost sites (*n* = 5); fresh plant material (including aquatic plants and fresh bog vegetation, *n* = 6); monospecies and mixed leaf litter (*n* = 5); freshwater and coastal sediments (*n* = 10); and one fossilized sample (lignite deposition, *n* = 1). To capture a wide diversity of OM, samples were collected across different climate zones in both hemispheres, from boreal to tropical environments, spanning a range of diagenetic ages and depths, from fresh OM found in plant material to highly diagenetically altered aquatic sediments and low-organic deposits. Additional details on sampling locations, environmental context, and sample handling are provided in Supporting Information (Table S1 and Text S1).

Fresh soil and sediment samples, except for the peat, were sieved through a stainless-steel mesh (2-mm pore size) at field moisture to remove larger particles and living plant residues, and then mixed thoroughly. Large roots and plant residues (>5 mm in diameter) were removed by hand from peat samples. Leaf litter and fresh plant material were cut into pieces smaller than 5 mm in length using scissors. All samples were stored in the dark at 4 °C until the start of incubation. Prior to incubation, all samples, except aquatic sediments, were adjusted to water-filled capacity to prevent moisture limitation, as detailed in Text S1; aquatic sediments were incubated as water-saturated slurries.

### Determination of the Bulk Chemical and Energetic Properties of Organic Matter

The molar-based concentrations of carbon (C), nitrogen (N), hydrogen (H), and oxygen (O) in the freeze-dried and powdered subsamples of all the materials were measured in triplicate on an elemental (CHN/O) analyzer (Costech 4010 Elemental Combustion System, Cernusco s/Nav., Italy). Elemental analysis was performed for C, H, and N separately from O, which required a separate pyrolysis step. Additional details on the analytical steps, including the analytical standards used for calibration and quality control, are given in Text S4. Subsamples were also acidified with 5% HCl prior to measurement to remove inorganic compounds. From this elemental analysis, the chemical formula of OM (C_a_H_b_N_c_O_d_), the C:N (carbon to nitrogen) ratio, and the oxidation state of carbon (*C*_*ox*_) were calculated, as described in Text S4. Because elemental oxygen was measured on bulk samples after acidification, the estimated oxygen content may include contributions from both OM and inorganic mineral phases (e.g., oxides and hydroxides). This source of uncertainty is expected to be greatest in 11 samples (i.e., samples from agricultural soils, aquatic sediments, and low-organic deposits) with low organic matter content (<5%) and is evaluated in Text S4. Thus, *C*_*ox*_ estimates for these samples should be interpreted with caution, although this does not affect the main conclusions of the study.

To determine the bulk energy content of solid samples, 200 mg of freeze-dried and ground material was placed into a Parr Isoperibol Oxygen Bomb Calorimeter, 6200 M20609 (Parr Instruments, Moline, Illinois, USA). The bomb calorimeter was calibrated and standardized using benzoic acid (Parr no. 3415, CAS. reg 65–85-0). Due to their high variation in OM content (2–95%), all samples were spiked with a known amount of standard mineral oil (VWR J217-500 mL mineral oil, light, white, high-purity grade) to achieve complete combustion, according to the calorimeter manufacturer’s manual. To calculate the energy of the sample, the contribution of the combustion aid was then subtracted from the total energy release. The calorific value was measured, and the specific energy content of the organic matter (kJ g C^–1^) was calculated. Measurements were not replicated because, in our previous experience with similar samples, the maximum variation of triplicates was <3.0%. Further details on the calculation, corrections, and assumptions of the method are provided in Text S2, where the calorimetric energy is described as the enthalpy of combustion (*ΔH*).

The Gibbs free energy of OM formation (*ΔG*_*f*_) was calculated as the difference between the enthalpy and entropy of OM formation (eq 1):

$$\Delta G^{f}=\Delta H^{f}-T\times \Delta S^{f}$$ 1

where: *ΔG*_*f*_ = the Gibbs free energy of ΟΜ formation (kJ mol^–1^); *ΔH*_*f*_ = the standard enthalpy of ΟΜ formation (kJ mol^–1^); *ΔS*_*f*_ = the standard entropy of ΟΜ formation (kJ K^–1^ mol^–1^); and *T* = the absolute temperature of the reaction (K). The enthalpy of OM formation (*ΔH*_*f*_) was calculated with bomb calorimetry, and the entropy of ΟΜ formation (*ΔS*_*f*_) was calculated from the elemental analysis (C, H, N, O).^30,38^ Further details and potential limitations of the calculations are provided in Text S5. Both specific energy and *ΔG*_*f*_ are expressed in kJ gC^–1^.

To characterize the structural composition and some basic functional groups related to the chemical composition of OM, freeze-dried and powdered samples were analyzed on a high-performance vacuum solid-state Fourier transform infrared (FTIR) spectrometer (VERTEX 80 V, Bruker, Germany). The ground sample (3 mg) was mixed with 297 mg potassium bromide (KBr, for IR spectroscopy, Uvasol®, Merck, USA) in a mortar, pressed into a 13 mm pellet, and measured covering a wavenumber range of 4,000–400 cm^–1^ with a spectral resolution of 4 cm^–1^. Anhydrous ethanol was used to clean the sample holder between samples. The OPUS 8.5.29 software (Bruker, Germany) was used to correct the baseline using the rubber-band method, and normalization was performed using the min-max method from the OPUS default option to account for differences in organic matter content. To minimize spectral interferences from minerals, only peaks within the 1,400–3,400 cm^–1^ range were evaluated, as low-organic deposits exhibited lower absorbance in this window. Peaks outside this range, particularly in regions where Si–O bonds overlap with polysaccharide signals (900–1,100 cm^–1^), were excluded from the analysis (Figure S9). The peaks used in the current analysis included the aliphatic CH_2_ stretching, the carbonyl C=O stretching in lipids, the O–H stretching in (hydro)­oxides of clay minerals, and the aromatic C=C stretching.^43,44^ The aromaticity index was calculated as the ratio of aromatic to aliphatic groups.^45^ The exact wavelengths and the functional group assignment are listed in Table S2.

### Determination of the Chemical Properties of Water-Extractable OM

To assess the properties of the water-extractable OM from all the samples, a water extract was prepared by mixing homogenized samples with Milli-Q water in a standard ratio of 1:10. The leachate was filtered through 0.7-μm glass microfiber, pre-combusted filters (GF/F grade, Whatman®, UK) and analyzed for dissolved organic carbon (DOC) and total dissolved nitrogen (TDN) on a TOC/TN analyzer (Shimadzu TOC-L/TNM-L, Kyoto, Japan). UV-Vis absorbance spectra were recorded on filtered samples across a wavelength range of 250 to 600 nm using a Thermo Fisher Scientific UV visible Spectrometer (Thermo Scientific Genesys 50, USA). From these spectra, three optical proxies were derived for the dissolved organic matter (DOM) characterization: the specific UV absorbance at 254 nm (SUVA_254_) as a proxy for DOM aromaticity,^19^ the exponential spectral slope of the log-transformed absorbance spectrum over the wavelength range of 250–465 nm, reflecting DOM molecular weight,^18^ and the 250/365 absorbance ratio as a proxy for nonaromatic and low molecular weight compounds.^46^ To characterize the chemistry of the water leaching from the soil, sediment, and litter, the pH of the water extracts was measured using a portable pH meter (Hanna HI991300; Woonsocket, Rhode Island, USA). Major anions (chloride, nitrate, phosphate, sulfate, acetate) and cations (sodium, ammonium, potassium, calcium, magnesium) were analyzed in 0.2-μm filtered samples using a Metrohm Ion Chromatography system (883 Basic IC Plus and 919 Autosampler Plus). A detailed description of the extraction and subsequent analyses is given in Text S6.

### Incubation Setup and Measurement of Headspace Gases (CO_2_ and O_2_)

To measure OM degradation rates (both CO_2_ production rate and O_2_ consumption rate), 3–5 g wet weight of the sample was placed into 60 mL Wheaton glass serum bottles (Sigma-Aldrich Corp., 1988) with ambient air. The bottles were sealed with airtight butyl rubber septa (Sigma-Aldrich Corp., 1988) and aluminum crimp caps (20 mm crimp caps, VWR). For each sample, 18 serum bottles were set up, allowing the sacrifice of three bottles at each of six time points. All the samples were incubated under aerobic conditions at 20 °C in the dark. Based on preliminary tests, sediments were incubated under gentle shaking on a horizontal shaker at 70 rpm to retain aerobic conditions.^47^ This was facilitated by adding 3–5 mL of Milli-Q water (1:1 ratio) to maintain their sediment character. All glassware was acid-washed and precombusted at 450 °C for 4 h.

We measured CO_2_ and O_2_ concentrations in the headspace of three independent replicate bottles on days 0, 4, 7, 14, 32, and 64 using a membrane inlet mass spectrometer (MIMS) (Bay Instruments, Easton, Maryland, USA) as has been previously described.^41^ Subsamples of the sieved, homogenized, and preconditioned materials were weighed at the start of the incubations, dried at 105 °C for 24 h, and then muffled at 550 °C for 4 h to determine moisture and organic matter (ash-free dry weight) content, respectively. We express mineralization as CO_2_ production and respiration as O_2_ consumption rates either normalized to sample dry weight (mass-specific, μmol gas gDW^–1^) or to sample OM weight (OM-specific, μmol gas gOM^–1^). The respiratory quotient (RQ), expressed as the molar ratio of CO_2_ production to O_2_ consumption, was calculated as the slope of the linear regression between CO_2_ production and O_2_ consumption over time. Details on the calculation of CO_2_ production and O_2_ consumption, including solubility corrections and carbonate system considerations, are provided in Text S3.

### Statistical Analysis

To evaluate the temporal evolution of CO_2_ production and O_2_ consumption and their relationship, we tested the fit of a linear model of gas concentrations over time and their correlation. Gas concentrations changed linearly in most samples. However, fresh plant material and leaf litter showed rapid O_2_ consumption within the first 7 days, reaching suboxic conditions, after which the relationship deviated from linearity. We applied segmented regression to identify the breakpoint at which CO_2_ and O_2_ evolution ceased to follow a linear trend and discarded time points where at least one of the gases ceased to change linearly. In two aquatic sediments, gas exchange was minimal, leading to negative values and poor linear fits; these samples were excluded from further analysis (Figures S1–S5). In the model, time was treated as a fixed effect, while replicate bottles, which were sacrificed at each time point, were considered independent measurements.

Differences among ecosystem types in non-normally distributed variables, including CO_2_ production and O_2_ consumption rates, RQ, bulk OM properties, and FTIR-based proxies of OM composition, were tested using the non-parametric Kruskal–Wallis test, with effect sizes (η^2^[H]) also calculated to quantify the proportion of variance explained by group differences. For variables showing significant differences among ecosystem types, post hoc pairwise comparisons were conducted using Dunn’s test with Benjamini–Hochberg (BH)-adjusted *p*-values. Because one ecosystem type (lignite) was represented by a single sample, it was excluded from the Kruskal–Wallis analysis but is included in all visualizations.

We also performed a partial least squares (PLS) analysis to determine which variables best predict OM decomposition rates. The importance of predictor variables in the model was determined by variable influence on projection (VIP) scores, where VIP scores ≥1 were considered highly influential, 1 > VIP ≥ 0.8 moderately influential, and <0.8 less influential. Cross-validation was performed to assess the model’s predictive power (Q^2^Y), and random permutation testing (100 permutations) of the response variable was used to assess the statistical significance of the estimated predictive power.^48^ Further information on the PLS analysis is provided in Text S7.

A linear regression model was fitted to relate potential OM decomposition rates to the variables identified as the most influential predictors in the PLS analysis. Model performance was checked visually by plotting residuals and calculating the root mean square error (RMSE). To evaluate multicollinearity of predictors, a Variance Inflation Factor (VIF) analysis was carried out between independent variables in the linear regression, where values greater than 5 were removed due to collinearity. Assumptions of normality and homoscedasticity were visually checked through the models’ residual diagnostic plots and also by Shapiro–Wilk and Breusch–Pagan tests, respectively. Statistical significance was assessed at a significance level (α) of 0.05.

All data analysis and plotting were conducted in R Statistical Software (Version 4.3.1; R: Core Team, 2023), except for the PLS analysis, which was done in SIMCA 17 (Umetrics, Umeå, Sweden).^51^

## Results and Discussion

### Potential OM Decomposition Rates and Variability in RQ across Environmental Sources

The rates of OM degradation, as measured by both CO_2_ mineralization and O_2_ respiration, varied significantly across the studied sources (Kruskal–Wallis test, *p* < 0.001; Figure 1), with a substantial effect size (η^2^[H] = 0.68–0.80, depending on the type of normalization). This variability is related to the diagenetic history of the OM, with fresh plant matter and leaf litter degrading most rapidly, followed by soils and sediments, with lignite degrading most slowly. This is consistent with previous studies in both terrestrial^4,49,50^ and aquatic ecosystems.^51–53^

**Figure 1:**
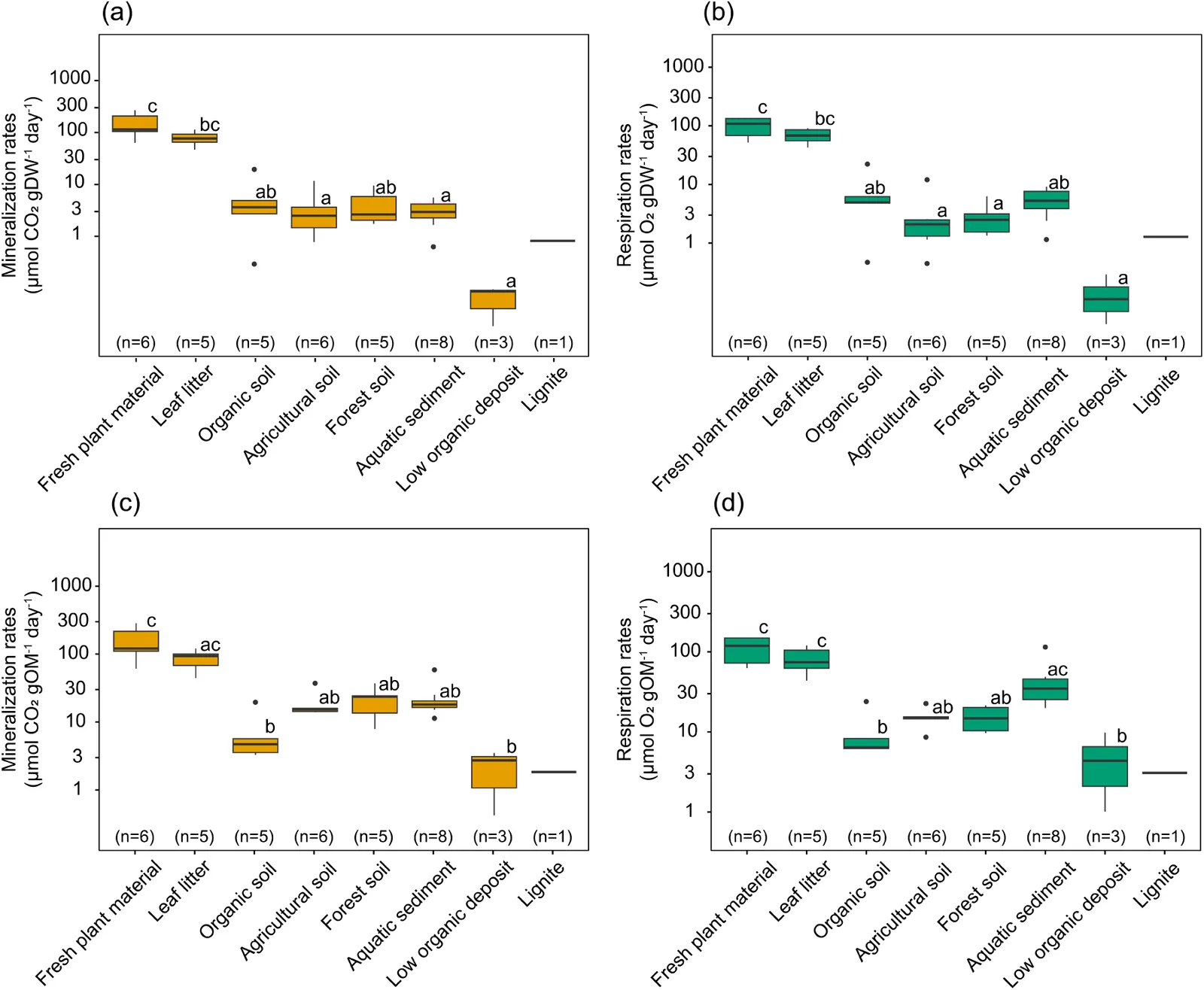
Mineralization and respiration rates across different OM sources. Each boxplot displays the median (central line), interquartile range (box), and whiskers extending to 1.5 times the interquartile range. Points represent outliers at each OM source. The upper panels (a, b) represent the mass-specific normalization method (gram of dry weight; gDW), while the lower panels (c, d) represent the organic matter-specific normalization method (gram of organic matter; gOM). The latter is calculated by loss of ignition when the freeze-dried sample is heated to 550 °C. Letters above boxplots indicate statistical groupings based on a post-hoc Dunn’s test following a Kruskal–Wallis analysis, where different letters represent significant differences between ecosystems (*p* < 0.05). Sample sizes (n) for each source type are indicated above the *x*-axis.

We found that patterns in decomposition rates depend on whether they are normalized to dry weight (DW) or to the weight of organic matter (OM). When normalized to dry weight (DW; mass-specific rates; Figure 1a, 1b), fresh plant material exhibited the highest mineralization (median ± standard error: 107 ± 32 μmol CO_2_ g DW^–1^ day^–1^) and respiration rates (111 ± 16 μmol O_2_ g DW^–1^ day^–1^), while lignite (0.8 μmol CO_2_ g DW^–1^ day^–1^, 1.3 μmol O_2_ g DW^–1^ day^–1^) and low organic deposits (0.1 ± 0.06 μmol CO_2_ g DW^–1^ day^–1^, 0.2 ± 0.6 μmol O_2_ g DW^–1^ day^–1^) showed the lowest rates. Other types of soils (organic, agricultural, and forest) and aquatic sediments showed intermediate but markedly lower values than fresh plant material and leaf litter. Similarly, when normalized to OM content (OM-specific rates; Figure 1c, 1d), fresh plant material (120 ± 37 μmol CO_2_ g OM^–1^ day^–1^, 121 ± 17 μmol O_2_ g OM^–1^ day^–1^) and leaf litter (94 ± 13 μmol CO_2_ g OM^–1^ day^–1^, 74 ± 14 μmol O_2_ g OM^–1^ day^–1^) maintained the highest degradation rates, followed by forest soils, aquatic sediments, and agricultural soils. However, OM-specific rates were substantially lower than mass-specific rates in organic soils, making them more similar to low organic deposits (3 ± 0.6 μmol CO_2_ g OM^–1^ day^–1^, 7 ± 6 μmol O_2_ g OM^–1^ day^–1^). To further explore the relationship between the two different normalization methods, we tested the correlation between DW and OM content across all samples. We found a negative relationship between DW and OM (R^2^ = 0.58, *p* < 0.001, *F*_(1,37)_ = 51.79; Figure S6), indicating that, while mass-specific and OM-specific decomposition rates generally follow similar trends, notable variation exists, suggesting that other factors beyond OM content may further influence the decomposition dynamics. Mass-specific rates capture the overall microbial activity within the sampled matrix and integrate both intrinsic OM properties and extrinsic controls, while OM-specific rates reflect the vulnerability of the OM stock itself. Thus, due to the wide variability in sample properties, the choice of normalization may affect interpretations, as previously discussed in studies of both soils and freshwater sediments.^54–56^ For this reason, both normalization approaches were considered in the PLS analysis to evaluate the influence of OM properties and other extrinsic variables on degradation rates.

### Microbial Degrading Performance and RQ Variability across Environmental Sources

To gain insight into microbial degradation performance, we estimated the RQ, defined as the ratio of CO_2_ production to O_2_ consumption. We found strong linear correlations between CO_2_ production and O_2_ consumption across the different ecosystems (Table S3 and Figure S5) indicating a strong stoichiometric coherence between the two rates. Respiration rates (μmol O_2_ g DW^–1^ day^–1^) were proportional to mineralization rates (μmol CO_2_ g DW^–1^ day^–1^) across the fresh plant material, the fresh litter, and the agricultural and forest soils, but substantially higher than mineralization rates in organic soils, lignite, aquatic sediments, and low-organic deposits, resulting in RQ < 1. These samples represent older OM that has undergone more extensive decomposition than fresh plant material and leaf litter. Overall, RQ differed significantly between the various sources (Kruskal–Wallis test, chi-squared = 27.62, df = 6, *p* < 0.001; Figure 2) with a large effect size (η^2^[H] = 0.70), indicating strong differences among environments and that a substantial proportion of the variance in RQ is explained by the environmental sources. The highest RQ was measured in fresh plant material (median ± standard error; 1.4 ± 0.2), followed by forest soils (1.2 ± 0.2), leaf litter (1.1 ± 0.1), and the agricultural soil (1.0 ± 0.1). Organic soils showed intermediate RQ values (0.7 ± 0.10) close to lignite (0.60, while the aquatic sediments and the low-organic deposits had significantly the lowest values (Figure 2) of all samples (0.4 ± 0.1 and 0.3 ± 0.1, respectively).

**Figure 2:**
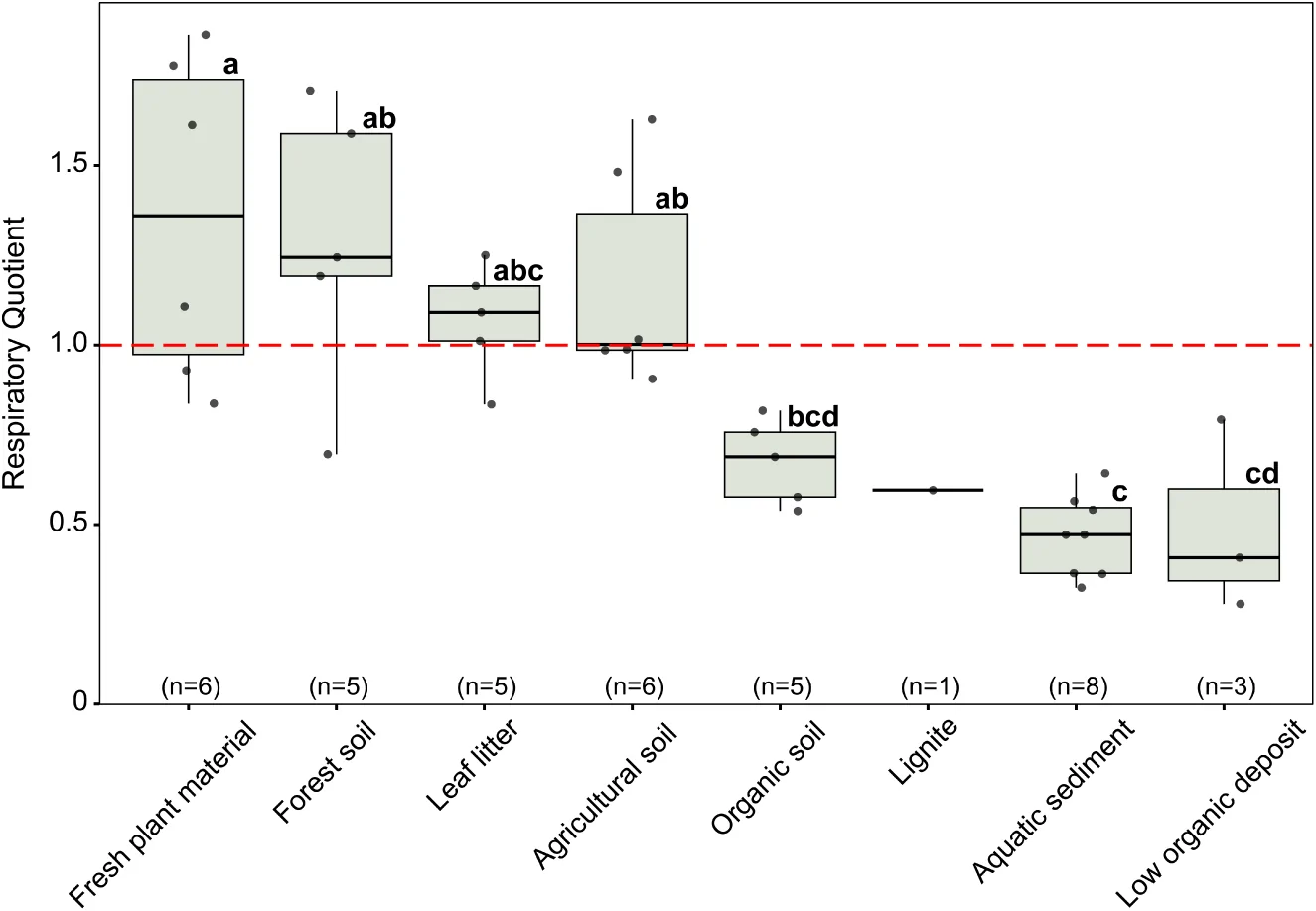
Respiratory quotient (RQ) variability across OM sources. Each boxplot displays the median (central line), interquartile range (box), and whiskers extending to 1.5 times the interquartile range. Points represent individual data values, showing variability within each ecosystem type. Letters signify the results of the post-hoc test (Dunn-test, *p* values adjusted with the Benjamini-Hochberg method) for the Kruskal–Wallis test and indicate significant differences across the different sources. Sample sizes (n) for each source type are indicated above the *x*-axis. The dashed red line indicates the assumed value of RQ = 1, which is frequently used in literature.

In general, based on the chemical properties of OM, RQ values ≥1 indicate the use of carbohydrate-rich or organic acid substrates undergoing complete oxidation. Similarly, RQ < 1 suggests catabolism of more reduced compounds.^40,57,58^ In our dataset, RQ varied significantly, spanning from 0.2 to 1.9 across the studied ecosystems. RQ ranged from higher values for fast-decomposing material (e.g., fresh plant inputs, leaf litter, and forest soils) to lower values where decomposition was slower, such as aquatic sediments, lignite, and low-organic deposits (Figure 2), consistent with preferential aerobic degradation of reduced, lipid-rich compounds.^59,60^ The lowest values were observed in low-organic deposits, likely due to low energy gain and oxygen-intensive degradation pathways. RQ was not correlated with specific energy or thermodynamic properties. This is consistent with recent findings by Hilman et al. (2022), who observed discrepancies between stoichiometrically calculated RQ based on the elemental analysis of OM and RQ derived from gas dynamics of degradation in soils and root systems.^61^ Similarly, in a recent cross-system study, a lack of correlation was observed between gas-measured RQ and the specific energy of bulk OM across different environments.^41^ This was explained by the fact that RQ might reflect the stoichiometry of the labile fraction metabolized by microbes instead of the elemental stoichiometry of the bulk OM pool. Together, these findings demonstrate that measured RQ may be the net mean value of several biogeochemical reactions and mainly reflects the fraction of OM that is microbially metabolized rather than the bulk OM pool’s stoichiometry and intrinsic energy density. Discrepancies between the stoichiometry of gas and bulk OM may also result from incomplete oxidation, where O_2_ is consumed without a corresponding release of CO_2_,^62,63^ or from microbial carbon retention in biomass.^34^

In our study, the RQ deviated substantially from the commonly assumed value of 1. Since RQ has been suggested to reflect the actual metabolic strategies employed by microbial communities, future studies should consider it as an indicator of microbial degradation performance for understanding the microbial processing of OM across environmental gradients, linking it with thermodynamic favorability, carbon- and energy-use efficiency, and respiratory behavior.^27,64–66^

### Relative Importance of Factors Influencing OM Microbial Decomposition

To evaluate the relative importance of factors controlling the microbial decomposition of OM, we applied a multivariate approach across a diverse set of environmental samples. By this, we assessed the relative explanatory power of several OM descriptors, including energetic, bulk stoichiometric, molecular, and environmental variables, in explaining the variation in decomposition rates. Two PLS models were used to identify predictors of both mass-specific and OM-specific decomposition rates. In both models, the CO_2_ and O_2_ rates were analyzed as response variables. The mass-specific model extracted two significant components, with an R^2^Y (variation in the response variable explained) of 0.83 and a Q^2^ (model predictivity) of 0.74. In comparison, the OM-specific model also extracted two significant components, but with lower R^2^Y (0.61) and Q^2^ (0.43), indicating a weaker model with lower predictive ability. Both PLS models were validated by using permutation tests (100 runs). They produced Q^2^ values of −0.27 (mass-specific) and −0.24 (OM-specific), along with small background correlations, with R^2^Y values of 0.15 (mass-specific) and 0.14 (OM-specific).

In the mass-specific model, the first PLS component explained 37% of the variance in OM decomposition rates (Figure 3a), and the second PLS component explained an additional 15%. Similarly, in the OM-specific model, the first PLS component explained 35% of the variance (Figure 3b), and the second PLS component explained 16%. The loading plots and the VIP values (Figure 3) of both models identified *ΔG*_*f*_, the O–H stretching intensity of (hydro)­oxides in clays (Clay), specific energy, aliphatic and lipid content, DOC, and the cation concentration of the leachate as highly influential explanatory variables of OM decomposition. The rates were positively correlated with all these variables, apart from the minerogenic O–H stretching intensity of the clays, which was negatively related to decomposition rates (Figure 3). The C:N molar ratio, carbon oxidation state (*C*_*ox*_), and DOM optical properties had only a moderate to weak influence on decomposition (Figure 3). Importantly, the mass-specific PLS model explained a greater proportion of variance in the response variable and showed higher predictive performance (Figure 3a) than the OM-specific model (Figure 3b). The suppressive effect of extrinsic controls (e.g., mineral matrices and cation interactions) may become pronounced, especially in low-organic deposits and sediments, when rates are OM-normalized. Accordingly, OM is protected against microbial degradation by association with minerals.^3,67,68^ This can be even more profound when scores are labeled by ecosystem (Figure S8). There, we observed a distinct clustering pattern separating samples with high OM content and corresponding high DOC concentration in the leachate (leaf litter, fresh plant material, and organic soils) from those with lower OM content and low DOC concentration in the leachate (forest soils, agricultural soils, aquatic sediments, and low organic deposits). The former group is associated with higher decomposition rates than the latter, which exhibits slower decomposition and a closer relationship to the O–H stretching intensity in clays.

**Figure 3:**
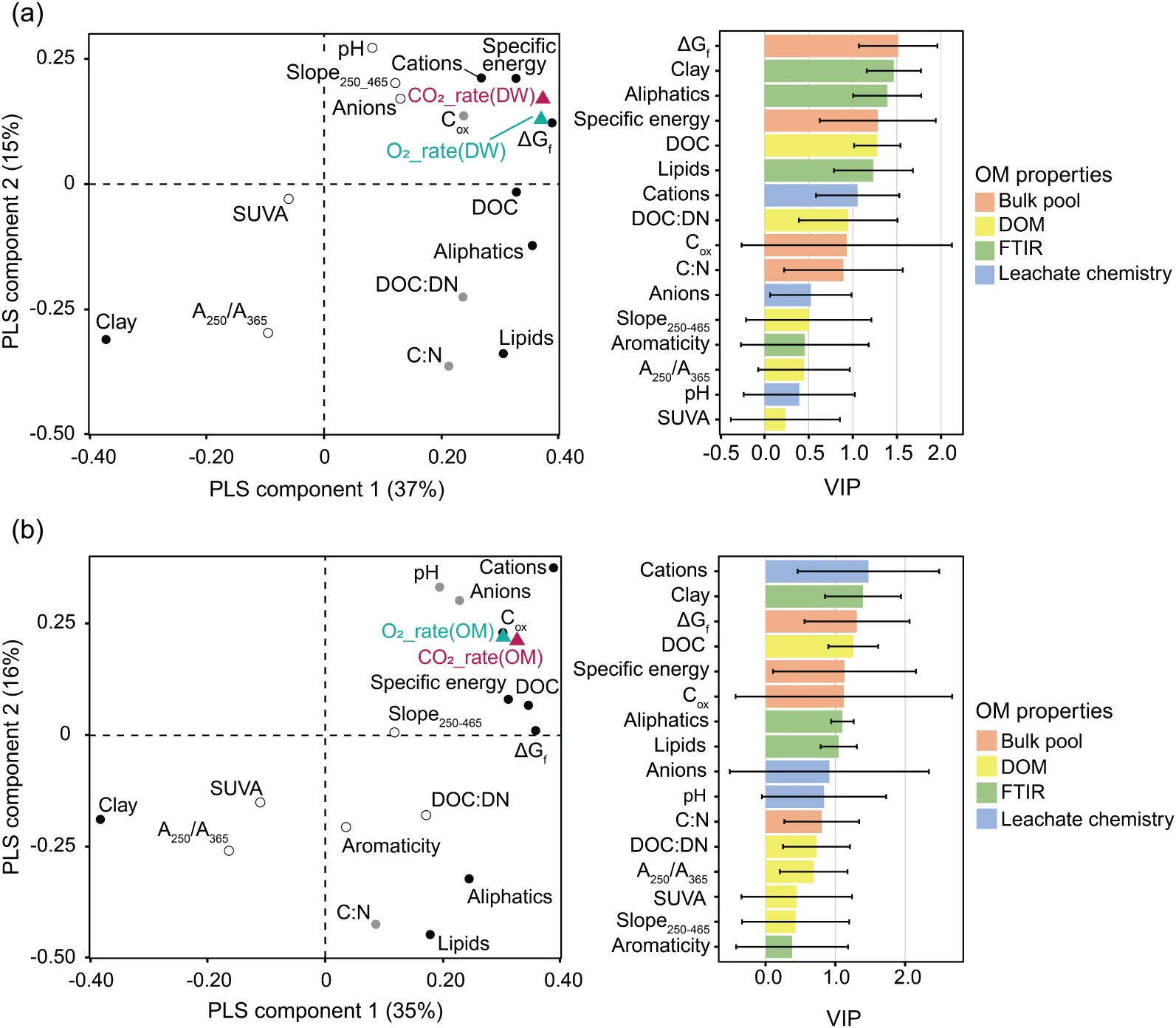
Partial least squares (PLS) analysis predicting mineralization and respiration rates based on organic matter (OM) bulk properties, FTIR proxies, leachate chemistry, and dissolved organic matter (DOM) characteristics. (a) Mass-specific CO_2_ and O_2_ rates, denoted as CO_2__rate (red triangle) and O_2__rate (turquoise triangle). (b) OM-specific CO_2_ and O_2_ rates. The left panels show PLS loading plots, where predictor variables are positioned based on their contributions to the first two PLS components (percent variation explained in parentheses). Filled black circles indicate highly influential variables (VIP ≥ 1), filled gray circles represent moderately influential variables (1 > VIP ≥ 0.8), and open circles represent less-influential variables (VIP < 0.8). The right panels display bar plots of variable importance in projection (VIP) scores, which quantify the influence of each predictor on the model. Error bars represent the standard error of VIP values. Predictor variables are color-coded by category: Bulk pool (orange), DOM (yellow), FTIR (green), and Leachate chemistry (blue).

Given the wide range of carbon contents across the studied samples, OM-normalized rates reflect the relative lability of the studied organic matter. However, the OM-normalized model did not reveal significant relationships with the tested explanatory variables. This likely reflects that bulk OM properties integrate both intrinsic and extrinsic controls, making it difficult to resolve consistent patterns across diverse samples and potentially masking the actual effects. Moreover, decomposition may also be constrained by the conversion of particulate organic carbon to dissolved organic carbon,^69^ suggesting that improved understanding of the energetic and chemical controls on the dissolved phase is needed to better explain OM decomposition dynamics.^24,70^

### Energetic Controls on OM Decomposition across Environmental Sources

Following the PLS analysis of the mass-specific decomposition rates, which demonstrated stronger predictive power than OM-specific rates, we identified *ΔG*_*f*_ as the strongest predictor. We then utilized this intrinsic OM property in a simple linear regression model (eq 2). We did not include the other strong predictor variables (related to intrinsic OM properties, specific energy, and aliphatic/lipid content) to avoid multicollinearity. Notably, moderate to strong positive and significant correlations were observed between *ΔG*_*f*_ and specific energy (R^2^ = 0.70, *p* < 0.001, *F*_(1,37)_ = 88.16, *n* = 39), as well as between *ΔG*_*f*_ and aliphatic content (R^2^ = 0.60, *p* < 0.001, *F*_(1,37)_ = 56.42, *n* = 39). The relationship between *ΔG*_*f*_ and specific energy reflects the dominant contribution of the enthalpic component to the Gibbs free energy of formation under aerobic conditions, where entropy effects are comparatively small and substrates with higher specific energy exhibit less negative Δ*G_f_* and greater energetic favorability for microbial oxidation.^71,72^

2 $$\ln\left(Decomposition\;rate\right)=a\times \Delta G^{f}+b$$

The simple linear model (Figure 4) explained a substantial proportion of the variation in mass-specific decomposition rates (R^2^ = 0.68, *p* < 0.01, RMSE = 1.28 (CO_2_) and 1.12 (O_2_) in ln units, *n* = 39). Predicted versus observed decomposition rates (Figure S10) and further model statistics (Table S3) are provided in the Supporting Information. To test if the observed relationship was driven by the single low organic deposit sample with the most negative *ΔG*_*f*_ and the slowest decomposition rates, we repeated the regression excluding this sample. The relationship remained still significant and of similar strength (R^2^ = 0.67, p < 0.01, *n* = 38; Table S4).

**Figure 4:**
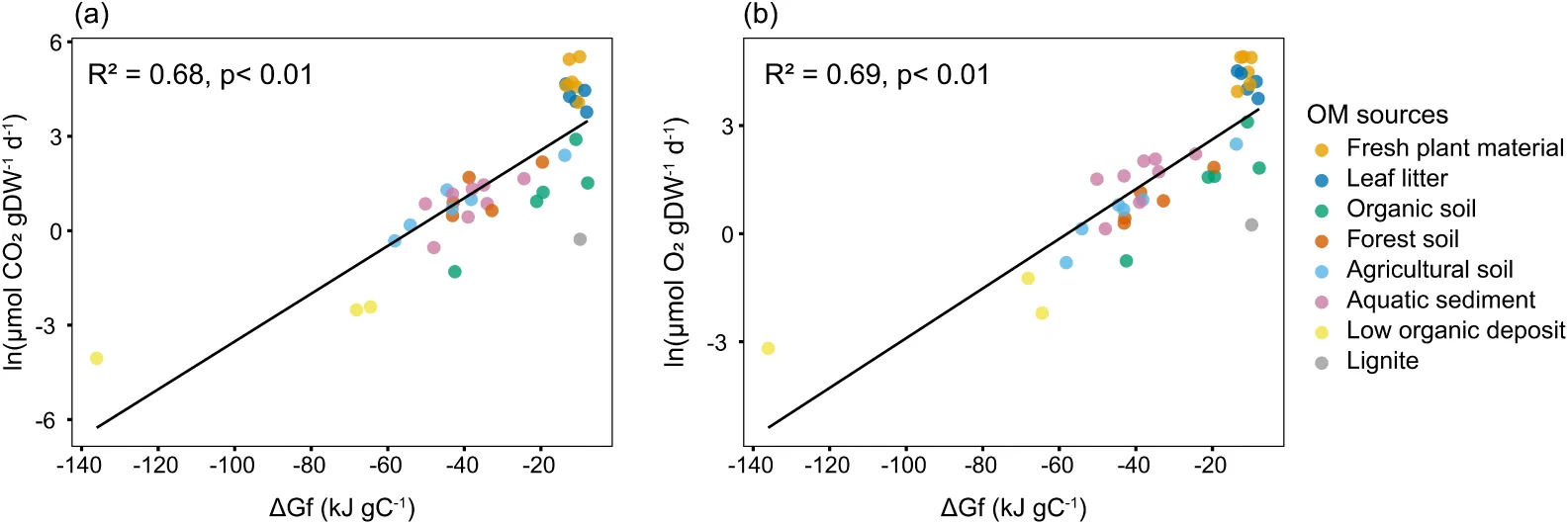
Relationships between the standard Gibbs free energy of formation (*ΔG_f_*, kJ gC^–1^) and respiration rates across studied materials. Panels (a) and (b) show mass-specific CO_2_ and O_2_ production rates, respectively. Respiration rates are expressed as the natural logarithm of μmol gas produced per unit dry weight (gDW^–1^) per day. Each point represents an individual sample, color-coded by OM source type. Linear regression lines are shown with corresponding R^2^ and *p* values.

Since the variation in DOC concentration in the leachate was coupled to the OM content of the samples, DOC concentration was also a highly influential predictor of OM decomposition. Therefore, including DOC as an explanatory variable in the linear regression model improved model prediction (R^2^ = 0.77, *p* < 0.01, Table S5) compared to the original model using only *ΔG*_*f*_ as a predictor (R^2^ = 0.68, *p* < 0.01; Figure 4; Table S3). The collinearity between the predictors *ΔG*_*f*_ and DOC was low (variance inflation factor; VIF < 3), well below the threshold of 5, suggesting no collinearity concerns. However, we decided not to incorporate DOC in the final model (*eq 2*) to simplify it. DOC concentration is highly affected by the extraction and filtration methods and needs to be measured shortly after extraction,^73^ whereas *ΔG*_*f*_ can be measured in dry samples using data from elemental analysis and bomb calorimetry at any time. Thus, to make the model as widely applicable as possible, we opted not to use DOC as a predictor. Model equations and performance that include DOC as a predictor variable are available in Table S5 for comparison.

Our main hypothesis, that the energetic properties of OM can predict degradation rates, was strongly supported. The PLS models (Figure 3), as well as a linear regression, revealed that *ΔG*_*f*_ is a strong predictor of decomposition (R^2^ = 0.68), as degradation rates were exponentially related to *ΔG*_*f*_ (Figure 5). This highlights the importance of intrinsic thermodynamic stability in determining OM decomposability across diverse environmental substrates.^27,30,71,74^ Organic matter with highly negative *ΔG*_*f*_ values is more thermodynamically stable and yields less energy upon oxidation, making it less favorable for microbial catabolism.^65^ In contrast, more positive *ΔG*_*f*_ values indicate OM that is thermodynamically less stable and requires less energy to break down into its end products (e.g., CO_2_ and H_2_O), making it easier to oxidize and more energetically rewarding for microbes. Leaf litter and fresh plant material had high specific energy and a more positive *ΔG*_*f*_ (∼−15 kJ gC^–1^), comparable to glucose (−12.7 kJ gC^–1^), reflecting the abundance of labile carbohydrates. In contrast, specific energy was lower and *ΔG*_*f*_ more negative (−30 to <−65 kJ gC^–1^) in OM from sediments and low-organic deposits, consistent with their higher thermodynamic stability and lower degradation rates (Figure 1).

**Figure 5:**
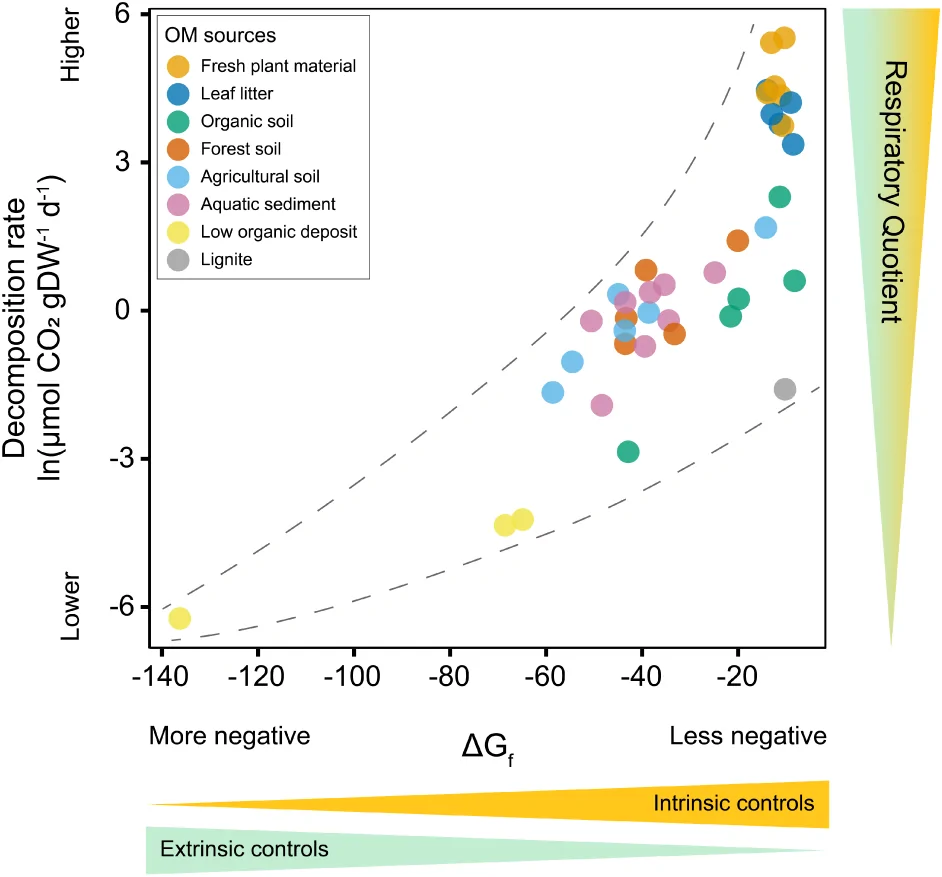
Conceptual illustration of controls on organic matter decomposition across the studied OM sources. The top panel illustrates the relationship between decomposition rates (CO_2_ production rate) and Gibbs free energy of OM formation (*ΔG_f_* in kJ g C^–1^), showing that substrates with less negative *ΔG_f_* values are associated with higher decomposition rates, while those with more negative *ΔG_f_* values decompose more slowly. Colored points represent different ecosystem types, as indicated in the legend. The bottom panel summarizes the relative contributions of intrinsic and extrinsic factors in controlling decomposition. Intrinsic controls (yellow) include indicators of energy availability and substrate composition, including specific energy and aliphatic content. Extrinsic controls (green) is the (hydro)­oxides content in clay minerals. The respiratory quotient (RQ) variability reflects microbial metabolic patterns and follows a similar trend to decomposition rates, which are affected by both intrinsic and extrinsic parameters across the studied environments. The data points in the biplot represent measured values on linear scales. Dashed lines indicate the bounds of the observed data points.

Compounds with greater structural complexity and lower entropy have more negative *ΔG*_*f*_, which explains why they are harder to degrade and less energetically favorable for microbes.^75^ Accordingly, OM with a higher aromatic content or stronger mineral association is generally considered recalcitrant.^30,76–78^ Thus, the stability inferred from *ΔG*_*f*_ may, in itself, act as a barrier to decomposition, independent of mineral protection. However, sorption to clays and metal oxides and physical occlusion in aggregates are well-documented mechanisms that limit microbial access and enzymatic degradation.^3,4,79,80^ This raises a central question: is slow decomposition in low organic deposits driven more by intrinsic thermodynamic constraints or extrinsic mineral protection? Our data suggest both mechanisms (thermodynamic stability and mineral protection) are at play, but distinguishing their relative importance remains challenging. Where *ΔG*_*f*_ is highly negative, even in the absence of strong mineral association, decomposition may still be inhibited, pointing to intrinsic thermodynamic control. Conversely, protection might dominate if OM with moderate *ΔG*_*f*_ values is stabilized due to mineral interactions.

Similar to *ΔG*_*f*_, specific energy decreased from fresh material to soils and sediments, reaching the lowest values in low organic deposits (Figure S11). The specific energy of OM correlated positively with decomposition rates (Figure 3a), supporting previous findings that heterotrophic microorganisms preferentially decompose high-energy OM, leaving behind lower-energy, more persistent material.^24,28,29,31,81^ This aligns with thermodynamic properties, which suggest that compounds rich in C–H bonds yield more energy upon oxidation and are thus more thermodynamically favorable for microbial degradation.^27^ In our data, aliphatic and lipid contents were among the strongest predictors of decomposition rates (Figure 3a). This supports the idea that high-energy functional groups are preferentially lost during degradation. Accordingly, Barré et al. (2016) showed that long-term bare fallow soils accumulate OM with lower energy content, as microbes preferentially consume high-energy material.^31^ Similarly, in our study, persistent OM in sediments and low organic deposits showed lower content of C–H bonding in aliphatic compounds (Figure S12) and specific energy (Figure S11), consistent with microbial selection against C–H-rich, energetically dense substrates. These findings support long-standing theories of OM diagenesis and microbial energy optimization.^82,83^ The pattern we found of more rapid degradation of OM with high specific energy is based on the bulk properties of OM, despite the fact that it is likely that degraders selectively utilize the components of the OM matrix with the highest specific energy.

However, an exception to the general pattern of thermodynamic control was observed in the lignite sample, which exhibited high specific energy values and more positive *ΔG*_*f*_ despite having only intermediate decomposition rates. Lignite also showed high specific energy and relatively less negative *ΔG*_*f*_ values, yet its OM was rich in both lipids and aromatic compounds. This unique combination likely represents an extreme case of OM with a complex macromolecular structure that is energetically rich but still less accessible. The co-occurrence of energetically favorable traits with structural resistance may limit decomposition rates despite high energetic values. Importantly, this exception does not undermine the broader applicability of our suggested framework, but instead highlights that exceptionally complex OM types, such as lignite, may behave differently from typical natural substrates found in terrestrial and aquatic ecosystems.

Our results reveal a clear thermodynamic and bioenergetic gradient in OM composition and degradability. While we use *ΔG*_*f*_ as a proxy for OM stability, activation energy (*Ea*), which has been extensively used in previous studies, may also reflect degradability, representing kinetic barriers to decomposition.^29,71,84^ These two perspectives are complementary: *ΔG*_*f*_ reflects the thermodynamic energy status of the OM pool, while *Ea* captures the energy required to initiate its degradation. OM can be structurally simple (low *Ea*) but thermodynamically stable (very negative *ΔG*_*f*_), particularly when protected by minerals. In such cases, microbes face low energetic returns despite intrinsically low *Ea*, resulting in slow decomposition. Supporting our findings, a bioenergetic framework in soils suggested that mineral-associated OM may contain chemically labile compounds (e.g., O-alkyl C, fatty acids) that might remain undecomposed due to physical protection.^29^ While we did not measure *Ea*, our findings imply that slow decomposition can be partly explained by chemical reactivity. The combination of low energetic favorability and mineral-associated physical protection likely contributes to the persistence of this OM. This supports the well-established concept that mineral-associated OM is selectively preserved due to a combination of physical protection and thermodynamic inaccessibility.^3^

### Toward a Generalized Energetic Framework of OM Degradation across Ecosystems

Considering the most influential factors on degradation across the studied environments, we propose a conceptual framework (Figure 5) of decomposition dynamics. This illustrates that decomposition patterns across OM sources can be simplified into a gradient driven largely by intrinsic energetic properties. OM with more positive *ΔG*_*f*_ values and a higher specific energy is associated with a higher aliphatic content and decomposes faster (e.g., fresh plant material and leaf litter). Various soil types and aquatic sediments show intermediate values for these variables and degrade more slowly. At the other end of the spectrum, OM with highly negative *ΔG*_*f*_ values and fewer C–H bonds tends to persist. Extrinsic factors, such as mineral associations (e.g., (hydro)­oxides content in clays) and microbial carbon metabolism (e.g., RQ), can further modulate this relationship by limiting or enhancing the effect of intrinsic OM properties, underlining the combined role of substrate composition and environmental conditions in controlling OM degradation.

This type of incubation experiment simplifies or excludes natural variability introduced by field conditions (e.g., soil heterogeneity, fluctuating temperature, water availability, redox dynamics, fresh inputs such as root exudates, microbial interactions, and solute transport), in particular, when samples with widely different in situ habitat structures and biotic interactions are compared. However, as exemplified here, they enable mechanistic insights beyond what is possible in the field and may shed light on patterns that are general across environmental sources.

Our study provides evidence that OM decomposition across natural sources is influenced by thermodynamic and energetic constraints, with Gibbs free energy of formation (*ΔG*_*f*_) emerging as a robust predictor of microbial mineralization and respiration rates. This energetic perspective presented here could be used to improve Earth system models. While current models typically rely on empirical decay constants for predefined OM pools,^33,85^ thermodynamic/energetic parameters can offer a more mechanistic and dynamic representation of OM turnover and enable linking of terrestrial and aquatic systems,^86,87^ with a holistic, ecosystem-independent perspective on OM decomposition. Most importantly, since chemical and energetic properties may capture different aspects of OM, incorporating both can provide a more complete view of OM reactivity across environmental sources.^31,88^

## Supplementary Material



## Data Availability

The data used in this study are available online at the Zenodo repository (10.5281/zenodo.18008474).

## Notes

Sample descriptions; metadata for sampling sites, sample nature, and selected OM properties (**Table S1**); detailed descriptions of sample collection and pre-incubation moisture conditioning (**Text S1**); links between bomb calorimetric energy and enthalpy of combustion (**Text S2**); carbonate system considerations and pH adjustment for headspace pCO_2_ measurements (**Text S3**); calculation of carbon oxidation state (*C*_*ox*_) and evaluation of non-organic oxygen contributions (**Text S4**); calculation of the Gibbs free energy of formation of organic matter (*ΔG*_*f*_) (**Text S5**); further details for the determination of water-extractable organic matter properties (**Text S6**); FTIR functional group assignments (**Table S2**); visualization of the linear regressions of CO_2_ production and O_2_ consumption rates normalized to dry weight and organic matter content (**Figures S1–S5**); relationships among organic matter, dry weight, and carbon content across ecosystems (**Figures S6–S7**); partial least squares (PLS) model results (**Figure S8**); FTIR spectra grouped by environmental source (**Figure S9**); relationships between *ΔG*_*f*_ and decomposition rates (**Figure S10**); boxplots of bulk and FTIR-derived organic matter properties across sources (**Figures S11–S12**); and summaries of linear models predicting CO_2_ mineralization and O_2_ respiration rates as a function of *ΔG*_*f*_ and dissolved organic carbon (**Tables S3–S5**) (PDF)
