# Supplementary material for: Energetic Controls on Biodegradation of Organic Matter from Diverse Environmental Sources
Source: Environ Sci Technol. 2026 Sep 10;60(37):26464–75. doi: 10.1021/acs.est.5c18534 (PMC13614203; doi:10.1021/acs.est.5c18534)
Supplement: Supplementary file 1 [file es5c18534_si_001.pdf]

Environmental Science & Technology

Supporting Information for:

Energetic controls on biodegradation of organic matter from diverse environmental sources

Konstantinos-Marios Vaziourakis[1], Liam Heffernan[1,2], Elizabeth Jakobsson[1],*

Dolly Kothawala[1] and Lars Tranvik[1]

[1]Department of Ecology and Genetics/Limnology, Uppsala University, Norbyvägen 18 D, 752 36 Uppsala, Sweden

[2]Department of Earth Sciences, Vrije Universiteit Amsterdam, De Boelelaan 1085, 1081 HV Amsterdam, The Netherlands (current position)

[*]Corresponding author email: konstantinos.vaziourakis@ebc.uu.se

This file contains 32 pages with:

- Supporting Text S1-S6
- Supporting Figures S1-S12
- Supporting Tables S1-S5

Table S1. List of the samples included in the study, accompanied by a description of the sampled material, location (name, country, and coordinates), and selected sample properties. Organic matter content (OM, % dry weight) was determined by loss on ignition (LOI), and organic carbon content (Org C, % dry weight) was measured using an elemental analyzer at the onset of the incubations. Specific energy (*SE*) and Gibbs free energy of organic matter formation (ΔG_f) are reported on a carbon basis (kJ g C-1). Not measured (n.m.)

ID	OM source	Sample Type	Description	Location (Latitude/Longitude)	OM (%)	Org C (%)	*SE* (kJ gC^{-1})	ΔG_f (kJ gC^{-1})	pH
3	Fresh plant material	Single-species fresh plant	Aquatic plant (lake), *Nuphar sp.*	Strandsjön, Uppland, Sweden (59°51'53.4"N/17°10'51.4"E)	89.6	43.8	39.2	-10.7	3.9
4	Fresh plant material	Single-species fresh plant	Aquatic plant (lake), *Iris sp.*	Strandsjön, Uppland, Sweden (59°51'53.4"N/17°10'51.4"E)	88.5	41.8	40	-12.6	5
14	Fresh plant material	Sphagnum-species fresh plant	Fresh growing sphagnum at the surface of thermokarst bog	Suossjavri, Finnmark, Norway (69°23'02"N/24°15'27"E)	96.7	44.3	40.4	-10.2	5.6
23	Fresh plant material	Single-species fresh plant	Aquatic plant (lake), *Potamogeton sp.*	Strandsjön, Uppland, Sweden (59°51'53.4"N/17°10'51.4"E)	88.1	40.8	41.8	-9.9	7.8
28	Fresh plant material	Single-species fresh plant	Fresh leaves collected from the sacred fig (*Phicus religiosa*)	Uppsala University Botanical Garden, Uppland, Sweden (59°51'02.3"N/17°37'41.3"E)	82.7	41.4	40.6	-13.4	7.6
37	Fresh plant material	Sphagnum-species fresh plant	Fresh growing sphagnum at the surface of thermokarst bog	Riba, Lappland, Sweden (68°28'37.1"N/21°00'25.7"E)	94.7	44.1	39.9	-11.9	4.6
5	Leaf litter	Single-species litter	Leaf litter (park), *Quercus robur*	Engelska Parken, Uppland, Sweden (59°51'16.3"N/17°37'41.5"E)	89.0	46.9	40.4	-10.8	5.4
6	Leaf litter	Single-species litter	Leaf litter (park), *Alnus glutinosa*	Bouleråker Boulebana, Uppland, Sweden (59°50'28.6"N/17°39'22.6"E)	92.0	48.7	42.1	-8.6	4.2
7	Leaf litter	Single-species litter	Leaf litter (mixed coniferous/deciduous forest), *Acer platanoides*	Stabbyskogen, Uppland, Sweden (59°50'38.4"N/17°37'32.9"E)	87.3	43.2	40.6	-13.5	6.7
26	Leaf litter	Single-species litter	Fallen needles, under Macedonian pine (*Pinus peuce*)	Uppsala University Botanical Garden, Uppland, Sweden (59°51'02.3"N/17°37'41.3"E)	97.6	49.6	41.6	-8.0	4.2
27	Leaf litter	Mixed-species litter	Mixed-species tropical dipterocarp forest leaf litter (forest floor)	Sukok Jungle, Brunei Darussalam (4°53'48.7"N/114°58'31.9"E)	71.3	43.9	40.5	-12.5	5.6
1	Forest soil	Soil; Organic O and A horizon	Mixed coniferous/deciduous forest (0–15 cm)	Fiby Urskog, Uppland, Sweden (59°53'00.5"N/17°21'05.0"E)	10.4	5.3	31.8	-42.9	4.8
2	Forest soil	Soil; Organic O and A horizon	Mixed coniferous/deciduous forest (0–10 cm)	Håga, Uppland, Sweden (59°49'41.8"N/17°34'19.6"E)	14.6	7.8	39.8	-38.8	5.2
22	Forest soil	Soil; Organic O horizon with pyrogenic OM	Burnt mixed coniferous/deciduous forest (0–5 cm), charcoal	Sala, Västmanland, Sweden (59°51'39.1"N/16°15'02.1"E)	24.0	12.8	34.8	-32.8	4.8

24	Forest soil	Soil; Organic O and A horizon	Mixed coniferous/deciduous forest (0–10 cm)	Grimsö field station, Västmanland, Sweden (59°43'02.9"N/15°29'14.6"E)	65.1	34.2	40.5	-19.6	4.7
25	Forest soil	Soil; Organic O and A horizon	Tropical lowland rainforest (0–15 cm)	Sukok Jungle, Brunei Darussalam (4°53'48.7"N/114°58'31.9"E)	6.7	3.5	39.1	-43.1	4.5
8	Agricultural soil	Soil; Organic O and A horizon	Wheat field (0–15 cm)	Gamla Uppsala, Uppland, Sweden (59°53'45.7"N/17°37'55.2"E)	5.2	2.0	34.6	-58.2	6.8
9	Agricultural soil	Soil; Organic O and A horizon	Perennial grassland (0–15 cm)	Bergsbrunna, Uppland, Sweden (59°48'27.2"N/17°43'12.7"E)	9.8	4.5	36.8	-44.5	6.4
35	Agricultural soil	Soil; Organic O horizon	Garden soil enriched in roots of *Dicksonia sellowiana*	Uppsala Botanical Garden, Sweden (59°51'02.3"N/17°37'41.3"E)	96.8	49.4	37.8	-7.8	4.9
29	Agricultural soil	Commercial horticultural bark mulch	Mix of sorted, non-fertilized bark from *Pinus sylvestris* and *Picea abies*	Rölunda (Natur täckbark, Sweden)	77.5	40.4	40.9	-13.7	6.2
30	Agricultural soil	Soil; Organic O and A horizon	Winter wheat field (0–15 cm)	Kungsängen, Uppland, Sweden (59°50'18.8"N/17°40'04.0"E)	7.6	2.9	36.0	-54.1	6.3
31	Agricultural soil	Soil; Organic O and A horizon	Pasture field (0–15 cm)	Håga, Uppland, Sweden (59°50'21.4"N/17°35'08.4"E)	13.6	7.1	32.1	-43.2	5.7
13	Organic soil	Soil; Histosol, organic A horizon	Sphagnum peatland (5–15 cm)	Jälla, Uppland, Sweden (59°55'14.8"N/17°42'13.6"E)	93.0	44.2	39.9	-10.8	6.3
15	Organic soil	Soil; Histel, organic A horizon	Permafrost soil layer (55–60 cm below active layer)	Storflaket, Abisko, Sweden (68°20'50"N/18°58'33"E)	59.6	33.5	40.0	-19.4	5.8
12	Organic soil	Soil; Gelisol, organic A horizon	Tundra/dwarf shrub tundra soil	Svalbard, Longyearbyen, Norway (78°10'18.2"N/16°03'43.6"E)	17.0	8.3	38.1	-38.2	5.7
36	Organic soil	Soil; Histosol, O horizon	Tropical peatland (45 cm below water surface)	Gambud, Brunei Darussalam (4°31'48.6"N/114°17'31.9"E)	7.6	4.0	39.5	-42.5	5.7
38	Organic soil	Soil; Histel, organic A horizon	Permafrost soil layer (75–90 cm below active layer)	Karlbotn, Finnmark, Norway (70°07'14"N/28°28'30"E)	77.4	31.1	40.4	-21.1	5.4
16	Aquatic sediment	Lake sediment	Eutrophic lake; 0–5 cm (surface layer)	Alstasjön, Uppland, Sweden (59°44'36"N/17°15'30"E)	9.3	4.1	35.7	-50.2	5.3
17	Aquatic sediment	Lake sediment	Humic lake; 0–5 cm (surface layer)	Stora Hålsjön, Uppland, Sweden (59°59'23"N/17°05'44"E)	46.1	26	40.8	-24.4	5.2
18	Aquatic sediment	Lake sediment	Oligotrophic lake; 0–5 cm (surface layer)	Långsjön, Uppland, Sweden (60°1′40″N/17°34′23″E)	12.4	10.1	21.8	-34	7.4
19	Aquatic sediment	Lake sediment	Eutrophic lake; 0–5 cm (surface layer)	Strandsjön, Uppland, Sweden (59°52'13.8"N/17°10'26.9"E)	20.1	11.1	41.7	-37.9	5.8
20	Aquatic sediment	Reservoir sediment	Reservoir; 0–5 cm (surface layer)	Rönne å reservoir, Skåne, Sweden (56°07'19.2"N/13°06'55.9"E)	24.6	13.1	40.5	-34.9	5.3
21	Aquatic sediment	Coastal sediment	Baltic Sea coastal sediment; 0–5 cm (surface layer)	Vedlösaviken, Uppland, Sweden (60°29'09"N/18°04'16"E)	1.0	0.6	29.1	-48.0	6

39	Aquatic sediment	Pond sediment	Botanical garden pond sediment	Uppsala Botanical Garden, Sweden (59°51'02.3"N/17°37'41.3"E)	9.2	5.3	33.1	-39.0	7.5
40	Aquatic sediment	Lake sediment	Eutrophic lake; 0–5 cm (surface layer)	Siggeforasjön, Uppland, Sweden (59°58'36.9"N/17°08'45.1"E)	21.2	8.9	36.6	-43.1	5.6
41	Aquatic sediment	Marine sediment	Marine sediment (5–5.45 m below seabed)	Brunei Bay, Brunei Darussalam (4°54'35.645"N/115°2'36.311E")	2.2	0.9	90.7	-58.3	5.2
42	Aquatic sediment	Coastal sediment	Coastal sandy sediment; 0–5 cm (surface layer)	Berakas beach, Brunei Darussalam (4°59'38.2"N/114°55'10.6"E)	0.3	0.05	n.m.	n.m.	7.3
11	Low organic deposit	Soil; Podsol, mineral B horizon	Coniferous forest (35–40 cm)	Grimsö, Sweden (59°43'02.9"N/15°29'14.6"E)	2.5	0.8	24.3	-64.5	6.1
33	Low organic deposit	Clay-rich soil layer exposed by erosion; parent C horizon	Miocene clay soil	Berakas beach, Brunei Darussalam (4°59'44.0"N/114°55'32.9"E)	3.0	1.0	19.3	-68.2	3.1
34	Low organic deposit	Clay-rich soil layer exposed by erosion; parent C horizon	Miocene lignite-bearing parent material	Subok, Brunei Darussalam (4°53'52.8"N/114°58'29.6"E)	4.1	0.7	4.9	-136.1	3.8
43	Lignite	Fossilised organic matter deposition	Miocene lignite in clay and sandy clay parent material	Subok, Brunei Darussalam (4°53'52.0"N/114°58'29.8"E)	41.5	51.2	40.4	-9.7	3.2

Text S1. Sample collection and pre-incubation moisture conditioning.

Lake and coastal sediments were retrieved with an Ekman grabber, representing the upper 5-10 cm. Marine sediment from Brunei Bay was collected using borehole drilling, and the analysed segment corresponded to 5.0–5.45 m below the seafloor. Leaf litter and fresh plant material were collected by hand and stored in plastic bags until further processing. For the soil samples, the upper 10-15 cm were removed by shovel to avoid the litter layer and to reach the soil horizon O up to the upper horizon A. The peat samples were collected using a Russian peat corer (5 cm inner-diameter, Eijkelkamp, Giesbeek, The Netherlands) or with a knife 10 cm under the moss vegetation. Surface vegetation from bogs was collected by hand, and the underlying peat was cored using the Russian corer. For sampling frozen permafrost, a steel pipe (inner diameter of 10 cm and 1.80 m long) was hammered down in 5 cm segments and pulled up again before being frozen to the substrate. The samples were gently pressed out of the pipe using a steel rod. All samples were kept cool and in the dark and immediately (within 24 hours) transported to the lab.

Before incubation, all samples except aquatic sediments, were preconditioned for 24 hours in rectangular aluminum foil trays (850 mL, Sanitas). Milli-Q (MQ) water was added to the samples at a 1:1 ratio (wet weight to MQ weight), and the trays were perforated with 0.8 mm needle holes to allow drainage of added MQ water. The trays were also covered with parafilm to prevent evaporation. The samples were left to drain for 24 hours at 10 °C to approach water field capacity. The field capacity was calculated by weighing the samples before and after drainage. Each sample was subsampled to determine the chemical properties of the water-extractable OM, and was also freeze-dried, ground, and stored in a desiccator until its chemical composition and energy content were analyzed, as described in the corresponding section of the main text.

Text S2. Description of the corrections and assumptions considered for the determination of the bulk energy content.

Bomb calorimetry was conducted under high oxygen pressure following the standard procedure described in the manufacturer's manual. Samples were spiked with a known amount of mineral oil as a combustion aid to ensure complete oxidation, and the contribution of the combustion aid was subtracted from the total heat release. Complete combustion was visually verified, and measurements indicating incomplete combustion (black soot in the residue) were discarded and repeated. The instrument measured the chemical energy of a sample by converting it to heat during combustion in an oxygen-saturated, closed system. Under isochoric conditions, this heat corresponds to the change in internal energy, and the combustion enthalpy is obtained. Albeit the complexity of OM, Korth et al., (2017) showed that the difference between combustion enthalpy and internal energy is minimal (<0.2%), validating the interpretation of the measured heat of combustion as enthalpy in this study as also previously used [2].

The formation of nitric and sulfuric acids during combustion can affect measured heat through solvation enthalpies. Nitric acid formation was accounted for using a fixed HNO_3 correction, as recommended by the manufacturer. This correction removes excess heat associated with the oxidation of atmospheric nitrogen in the combustion vessel, introducing only minor uncertainty. Sulfur content was measured independently with the same analyzer described in the methods section and was low across samples (<1%, with one sample of 1.35%). At these concentrations (<1–2%), the contribution of sulfur oxidation to the heat of combustion is negligible (on the order of tens of calories g^{-1}); therefore, no sulfur correction was applied according to our personal communication with the manufacturer.

Additional factors (e.g., temperature dependence of the calorimeter constant, compressibility work, and water vaporization enthalpy) can influence calorimetric measurements but are primarily relevant for high-precision studies of homogeneous compounds. These higher-order Washburn corrections, in combination with their evaluation using the NIST combustion calorimetry tool, were not applied due to the heterogeneity of the analyzed environmental samples. For these types of environmental samples (soils, sediments, biomass), standard methods (e.g., ISO 18125:2017; EN ISO 21654:2021) do not include these terms because their contribution is small relative to sample heterogeneity and analytical uncertainty. Therefore, these specific corrections would lead to negligible improvements relative to sample variability.

Analytical uncertainty was evaluated using replicate measurements (n = 5) of standard materials (glucose and marine sediment). Marine sediment was analyzed in a certified laboratory at SLU (Ultuna) and reanalyzed in our laboratory, while glucose (Sigma-Aldrich, St. Louis, MO, USA; G7021, ≥99.5%) was used as a commercial standard and compared to manufacturer specifications. Precision was in the range of 1-3%, while accuracy ranged between 2-8% relative to reference values. These uncertainties are small compared to the variability observed across environmental samples and are therefore not expected to influence the interpretation of thermodynamic patterns. Accordingly, the reported thermodynamic properties represent bulk descriptors of organic matter energetic state, and uncertainties associated with omitted corrections are small compared to variability across samples.

Text S3. Carbonate system and pH adjustment in measuring pCO_2 in headspace.

For all the samples, CO_2 produced and O_2 consumed by respiration were calculated as CO_2 increase and O_2 decrease in the headspace and the water phase, according to the ideal gas law and Henry's law, where coefficients for CO_2 and O_2 solubility in water were obtained from Sander, 2023. From the calculations, we excluded bicarbonate and carbonate ions because the initial pH of the samples was always < 7.3, except for three samples (sample nos. 18, 28, and 42) for which the pH was 7.59, 7.68, and 7.95, respectively, but decreased substantially during the incubation. Below pH 7.3 in freshwaters, an increase in CO_2 production will primarily increase dissolved CO_2 concentrations rather than the other carbonate system species (HCO_3^- and CO_3^{2-}; Polsenaere and Abril, 2012) if there is no change in alkalinity. This means that in incubations, when carbonate precipitation or dissolution is negligible [5,6], the change in dissolved inorganic carbon equals the change in CO_2. To measure pH during the incubation, two replicates at each time point were sacrificed after the gas measurements. For this, 5 g of wet weight of all the materials was mixed 1:10 with 20 mL of Milli-Q water. This mixture was shaken for 1 hour at 240 rpm and then centrifuged at 3,000 rpm for 15 minutes. The pH of the supernatant was measured with a Hanna mobile pH meter (Hanna HI991300; Woonsocket, Rhode Island, USA).

Text S4. Description of the analytic procedure for the CHN/O determination, calculation of carbon oxidation state (C_{ox}) and evaluation of non-organic oxygen contribution.

The samples were weighed in tin (12mm x 6mm, product number C11590.200P, OEA labs) or silver (8mm x 5mm, product number D2308, Elemental Microanalysis) capsules for NCH and O analysis, respectively, on a Sartorius SE2 microbalance (d=0,1µg).

Analysis was performed on a Costech 4010 Elemental Combustion System in either combustion (CHN) or pyrolysis (O) mode using commercially available pre-packed reaction tubes

and separation columns. Helium was used as the carrier gas and instrument settings were set following the general operating parameters described in the manual.

Calibration curves were established using acetanilide (product number B2114, Elemental Microanalysis) for O and with sulphanilamide (product number, B2036, Elemental Microanalysis) for CHN. Quality control was performed by analyzing the two Organic Analytical Standards Soil Standard Peaty (product number B2176, Elemental Microanalysis) (for CN) and EDTA (product number 2033, Elemental Microanalysis) (for CNHO).

The oxidation state of carbon (C_{ox}) is used to describe the oxidation state of bulk organic matter in environmental samples, with values ranging from –4 for methane (the most reduced form of carbon) to +4 for carbon dioxide (the most oxidized form), calculated from the neutral molar percentages of C, H, N, and O measured on the elemental analyzer, and based on the following *equation S1* [7]. This equation assumes that the oxidation states of non-carbon species are the following: $H_{ox} = 1$, $O_{ox} = -2$, and $N_{ox} = -3$, and neutrality of the organic molecules. We also omitted S and P from the calculations, since they contribute marginally to the oxidation state compared to the dominant redox-active elements.

$$Cox = \frac{2[O] - [H] + 3[N]}{C} \qquad (equation\ S1)$$

To evaluate the potential contribution of inorganic oxygen to elemental oxygen measurements, we summarize here a validation experiment conducted as part of the MSc thesis of Pauline Balk (2024). Additional methodological details are available in [8]Balk (2024). The validation experiment was conducted using organic matter gradients consisting of oak leaf litter mixed with $CaCO_3$ and goethite at known proportions. Carbon, hydrogen, and nitrogen closely matched their expected values (<1.3% deviation), whereas oxygen showed larger deviations, particularly for the mineral standards, where the measured oxygen content was 29% lower for $CaCO_3$ and 28% lower for goethite than expected (Table 4 in M.Sc. thesis). This suggests that oxygen associated with mineral phases was recovered much less efficiently than oxygen associated with OM and that the bulk elemental oxygen measurements are weighted toward oxygen associated with the organic fraction.

To assess whether this limitation influences environmental samples, bulk C_{ox} values were compared with C_{ox} values calculated after NaOCl treatment, which estimates the contribution of the inorganic fraction. Despite the uncertainty associated with inorganic oxygen measurements, no significant differences between bulk and corrected C_{ox} were observed for any environmental sample apart from the agricultural soil (Figure 3 and Table 6 in the MSc thesis), indicating that, for the samples investigated here, the contribution of inorganic oxygen was insufficient to substantially alter the estimated C_{ox}. These results indicate that the present approach cannot eliminate interference from inorganic oxygen, particularly in mineral-rich samples. More rigorous separation of organic and inorganic phases (e.g., HF pretreatment before elemental analysis, NaOCl-based correction) could further reduce this uncertainty, although these methods are more laboratory-intensive and may themselves modify OM composition. Solid-state ^{13}C NMR can be an alternative method; however, its cost remains high and the measurement time quite extensive. Therefore, C_{ox} estimates with an elemental analyzer, especially for mineral-rich samples, should be interpreted with caution.

Text S5. Calculation of ΔG_f (Gibbs free energy of OM formation).

The ΔG_f was calculated based on the difference of entropy (ΔS_f) from the enthalpy of organic matter formation (ΔH_f) using *equation S2* [9]

$$\Delta G_f = \Delta H_f \; - T\Delta S_f \qquad \textit{equation S2}$$

where: ΔG_f = the Gibbs free energy of organic matter formation per gram organic matter (kJ mol^{-1}); ΔH_f = the standard enthalpy of organic matter formation (kJ mol^{-1}); ΔS_f = the standard entropy of organic matter formation (kJ K^{-1} mol^{-1}); and T = absolute temperature of the reaction (K). We normalized ΔG_f per gram of carbon (kJ g C^{-1}).

We calculate ΔH_f from the measured heat of combustion, bulk energy (kJ mol^{-1}), determined using bomb calorimetry, and Hess's law using *equation S3*

$$\Delta H_f = \Delta H_{f-CO2} + \Delta H_{f-H2O} \; - \Delta H_{f-C} \qquad \textit{equation S3}$$

where; ΔH_f = the standard enthalpy of organic matter formation (kJ mol^{-1}); $\Delta H_{f\text{-}CO2}$ = the standard enthalpy of CO_2 formation (kJ mol^{-1}); $\Delta H_{f\text{-}CO2}$ = the standard enthalpy of H_2O formation (kJ mol^{-1}); and $\Delta H_{f\text{-}C}$ = the measured bulk energy of organic matter combustion from bomb calorimetry (kJ mol^{-1}). We calculated $\Delta H_{f\text{-}CO2}$ and $\Delta H_{f\text{-}H2O}$ from Hess's law by multiplying the number of atoms in each sample found in CO_2 and H_2O by -393.5 and -285.8 kJ mol^{-1}, respectively[10] (National Institute of Standards and Technology (NIST), 2017).

The entropy of organic matter formation (ΔS_f) was estimated using measured elemental compositions and *equation S4*

$$\Delta S_f = S^{\phi}_{OM} - \textstyle\sum_{i=1}^{i} S^{\phi}_{i} \qquad \textit{equation S4}$$

where; ΔS_f = the standard entropy of organic matter formation (kJ K^{-1} mol^{-1}); S^{ϕ}_{OM} = the absolute entropy (J K^{-1} mol^{-1}) of the organic matter; S^{ϕ}_{i} = the absolute entropy (J K^{-1} mol^{-1}) of the element

(*i*). To determine S_i^{ϕ} , we multiplied the number of atoms of each element in the organic matter by the absolute entropy of the elements C (5.5), H (130.5), O (205), and N (191.5), all with the unit J K^{-1} mol^{-1}. We determined the absolute entropy of organic matter (S_{OM}^{ϕ}) from the heat capacity (C_{OM}^{ϕ}) and *equation S5*

$$S_{OM}^{\phi} = 1.1C_{OM}^{\phi} \qquad \textit{equation S5}$$

The absolute heat capacity (C_{OM}^{ϕ}) is calculated using *equation S6*

$$C_{OM}^{\phi} = \sum_{i=1}^{i} C_i^{\phi} n_i \qquad \textit{equation S6}$$

where; C_i^{ϕ} = the atomic heat capacity, which for C, H, N, and O are 7.53, 9.62, 11.3, and 16.74 J K^{-1} mol^{-1}, respectively (NIST, 2017), and n_i = number of atoms of element *i* in the organic matter.

Uncertainty in the estimation of ΔG_f can arise from both the experimental determination of combustion enthalpy and the approximation of the entropic term. Combustion was performed using bomb calorimetry under high oxygen pressure, with mineral oil used as an ignition aid, which is the best available standard to reach complete oxidation of the samples under standard operating conditions. Therefore, uncertainties associated with incomplete combustion are expected to be minimal. In contrast, the entropy term derived from elemental composition represents a simplified approximation and may not fully capture the molecular complexity and structural heterogeneity of natural organic matter. Future improvements could be achieved by incorporating structural and molecular-level information (e.g., solid state NMR) to better constrain entropy estimates. Additionally, mineral–organic interactions may influence measured combustion enthalpy, particularly in more minerogenic samples, potentially introducing deviations from the intrinsic energy content of organic matter. Such effects are expected to be less significant in organic-rich materials such as leaf litter, fresh plant material,

and organic soils. As such, ΔG_f should be interpreted as an integrative bulk descriptor of OM energetic state rather than an exact thermodynamic property. Despite these limitations, the consistency of observed relationships across diverse samples suggests that ΔG_f captures meaningful variation in OM energetics relevant to microbial decomposition processes.

Table S2. Functional group assignments of selected FTIR absorption peaks used in this study. The peaks, used in the current analysis, included the aliphatic CH_2 stretching, the carbonyl C=O stretching in lipids, the O–H stretching in (hydro)oxides of clay minerals, and the aromatic C=C stretching [11,12]. The aromaticity index was calculated as the ratio of aromatic to aliphatic groups [13].

**Peak wavenumber (cm^{-1})**	**Assignment**
2,920 and 2,850	Aliphatic CH_2 stretching vibrations
1,740	Carbonyl (C=O) stretching (lipid content)
3,620 and 3,700	O-H stretching vibrations of minerogenic (hydro)oxides
1,610 and 1,520	Aromatic C=C stretching (aromatic content)
$\frac{1{,}610\ and\ 1{,}520}{2{,}920\ and\ 2{,}850}$	Aromaticity index

Text S6. Water extractable organic matter properties.

Approximately 5 g wet weight of each sample, homogenized before the incubation, was mixed with 45 mL of MQ water, creating a 1:10 solution in 50 mL MQ-washed Falcon tubes. This mixture was shaken for 1 hour at 240 rpm and then centrifuged at 3000 rpm for 15 minutes. The supernatant leachate was collected for analysis as described below.

The pH of the water extractable solution derived from each sample was measured in the supernatant with a Hanna mobile pH meter (Hanna HI991300; Woonsocket, Rhode Island, USA). Dissolved organic carbon (DOC) and total dissolved nitrogen (TDN) concentrations were determined using glass microfiber filtered (GF/F grade, pore size of 0.7 µm, Whatman®, UK) water on a TOC/TN analyzer (Shimadzu TOC-L/TNM-L, Kyoto, Japan). The GF/F filters were pre-combusted at 450°C for 4 hours to remove remaining organics.

UV–Vis absorbance spectra (250 to 600 nm) of GF/F filtered water were measured in a 1-cm quartz cuvette using a Thermo-Fisher Scientific UV visible Spectrometer (Thermo Scientific Genesys 50, USA). The specific UV absorption coefficient at 254 nm ($SUVA_{254}$; L mg C^{-1} m^{-1}), a proxy for dissolved OM (DOM) aromaticity [14], was calculated as the ratio between A_{254} and the DOC concentration (mg C L^{-1}). The 250/365 absorbance ratio was used as a proxy for nonaromatic and low molecular weight compounds [15], where high values indicate a low degree of aromaticity and low molecular weight, and low values denote a high degree of aromaticity and high molecular weight. Moreover, the 250–465 nm slope was calculated as an additional optical parameter to characterize DOM composition. Similar to the 250/365 absorbance ratio, the spectral slope provides insight into the molecular weight and aromaticity of DOM, with steeper slopes indicating lower molecular weight and reduced aromatic content. In comparison, shallower slopes reflect larger, more aromatic molecules [16].

Anions and cations were measured in samples filtered through pre-rinsed 0.2-µm Acrodisc Supor hydrophilic polyethersulfone membranes (Pall Laboratory, Port Washington, New York, USA) and stored frozen immediately after extraction. Anions (Cl^-, NO_3^-, PO_4^{3-}, SO_4^{2-}, and acetate) were separated on a Metrosep A Supp 5 analytical column (150 × 4.0 mm) fitted with a Metrosep A Supp 4/5 guard column at 0.7 mL min^{-1} using a carbonate eluent (3.2 mM Na_2CO_3 + 1.0 mM $NaHCO_3$). Cations (Na^+, NH_4^+, K^+, Ca^{2+}, and Mg^{2+}) were separated on a Metrosep C4 column (250 × 2.0 mm) with a Metrosep C4 guard column with an eluent of 1.7 mM nitric acid and 0.7 mM dipicolinic acid and a flow rate of 0.2 mL min^{-1}. The analyses were conducted on a Metrohm IC system (883 Basic IC Plus and 919 Autosampler Plus).

Text S7. Partial Least Square (PLS) analysis on the variables predicting decomposition rates.

PLS analysis was performed to determine which variables best predict OM decomposition rates. From a statistical perspective, PLS was selected because it is designed to handle situations where predictors are numerous and potentially collinear. PLS constructs orthogonal latent components that maximize the covariance between predictors and response variables, making it particularly appropriate for datasets in which variables such as chemical composition and energetic metrics may share some covariance. Here, we would like to see how variables cluster together in the ordination space. In this context, including both sets of variables (energetic and chemical OM descriptors) enabled us to evaluate their relative importance within a single predictive framework rather than assuming a predefined causal structure.

PLS analysis was especially suitable for this data set as it is insensitive to co-relationships between the predictor variables. Highly skewed predictor variables (skewness >2.0 and/or min/max ratio < 0.1) were log-transformed, and the response variables (mineralization and respiration rates) were also log-transformed to increase model performance. All predictor variables were mean-centered and scaled to unit variance before analysis. We reduced the

variables by grouping the cations (Na^+, NH_4^+, K^+, Ca^{2+}, and Mg^{2+}) and anions (acetate, Cl^-, NO_3^-, PO_4^{3-}, and SO_4^{2-}). RQ was excluded from the PLS models due to its dependency on the gas rates used as response variables. For better visualization and direct comparison between models, we adjusted the sign orientation of the PLS loadings while preserving the relative relationships among variables. This does not alter the interpretation, as PLS loadings have arbitrary sign conventions. The original, unadjusted loadings are provided below in the (**Figure S8**) for reference.

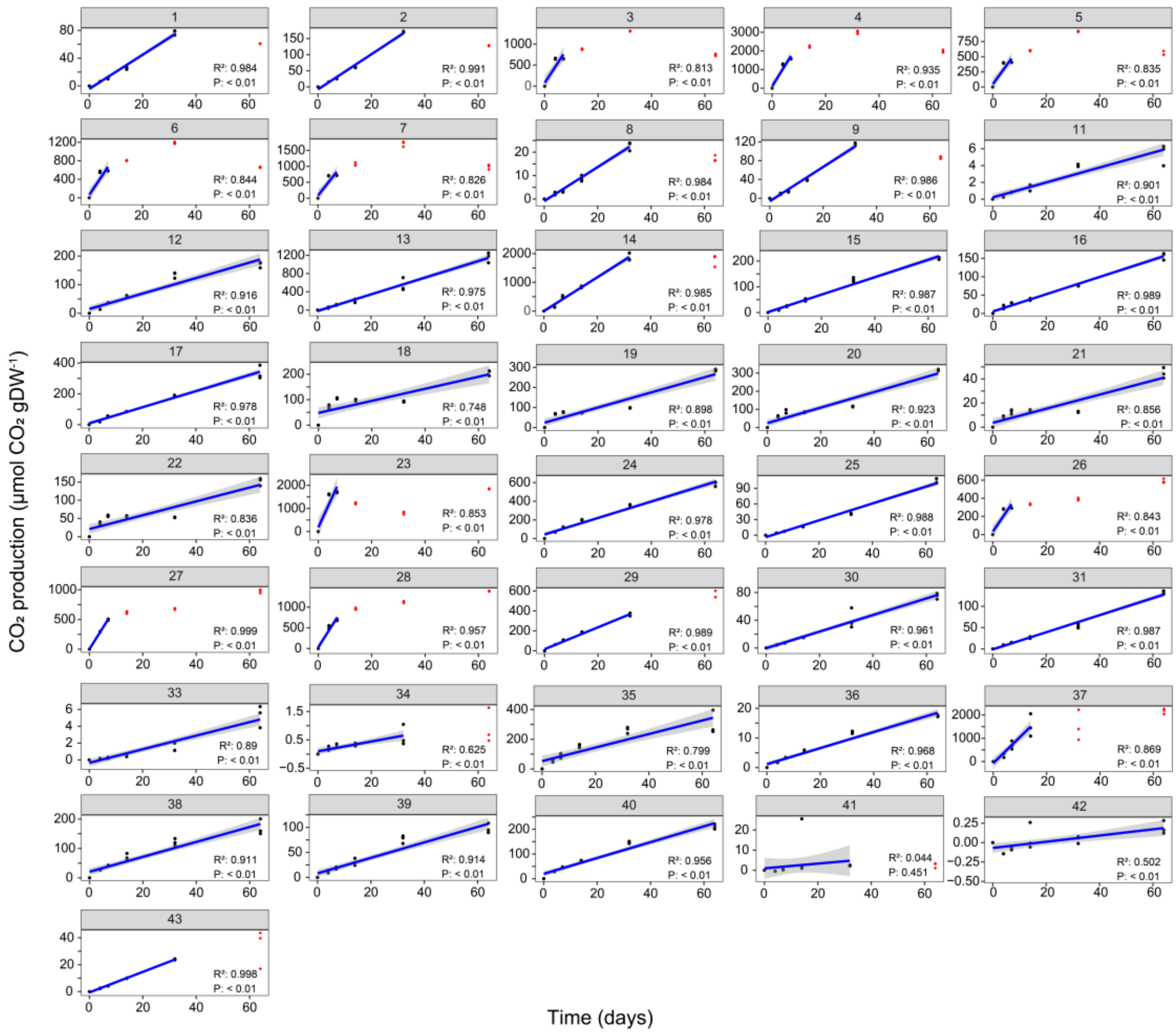

Figure S1. Linear regression of CO_2 production (µmol CO_2 g dry weight^{-1}) over time (days). Black points represent data included in the regression analysis, while red points indicate excluded time points where CO_2 and/or O_2 concentrations deviated from linearity based on segmented regression analysis, suggesting a change in gas dynamics. Each panel corresponds to a different sample, with R^2 and p-values reported for the fitted linear models. Shaded areas around the regression line represent 95% confidence intervals.

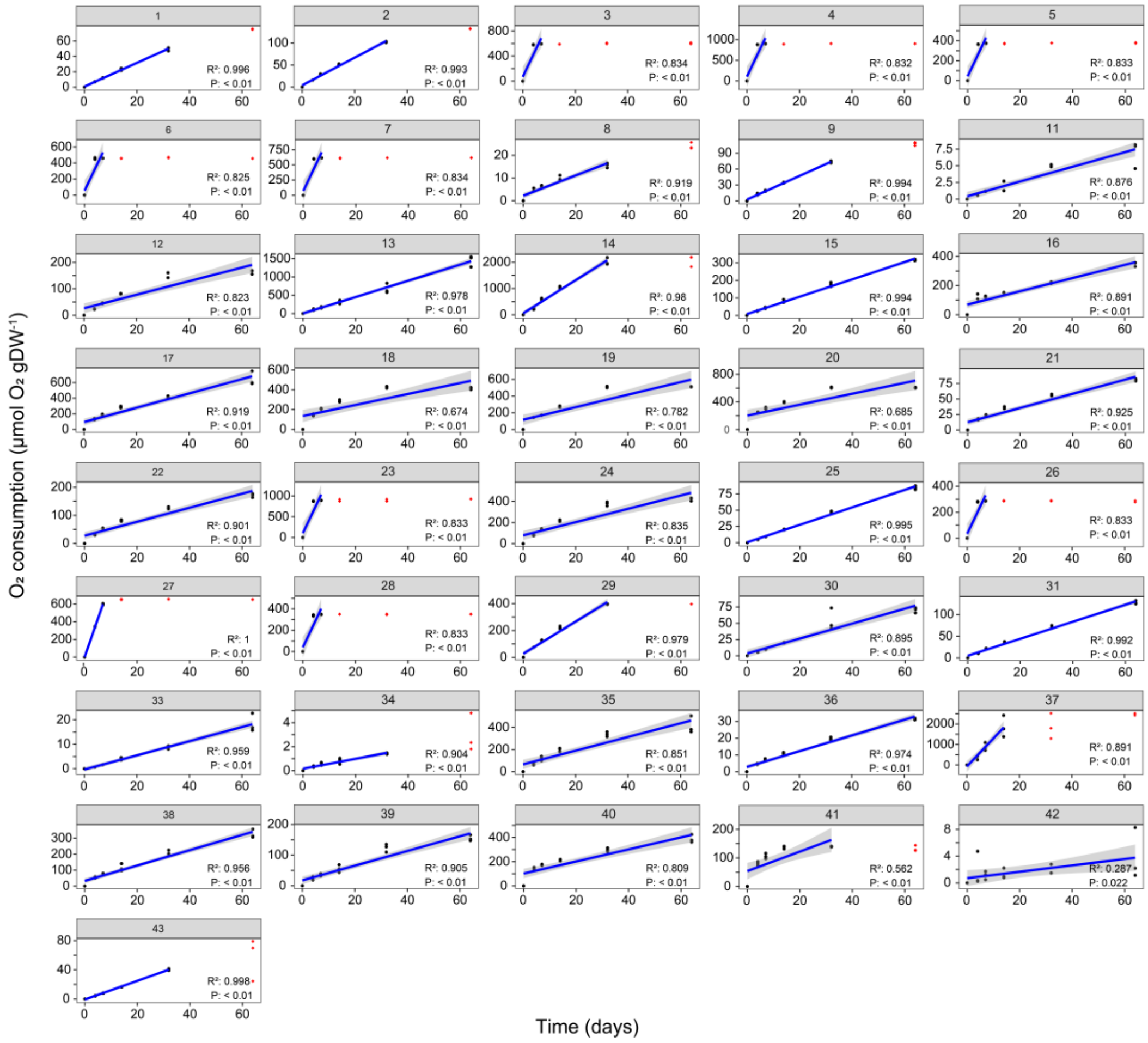

Figure S2. Linear regression of O_2 consumption (µmol O_2 g dry weight^{-1}) over time (days). Black points represent data included in the regression analysis, while red points indicate excluded time points where CO_2 and/or O_2 concentrations deviated from linearity based on segmented regression analysis, suggesting a change in gas dynamics. Each panel corresponds to a different sample, with R^2 and p-values reported for the fitted linear models. Shaded areas around the regression line represent 95% confidence intervals.

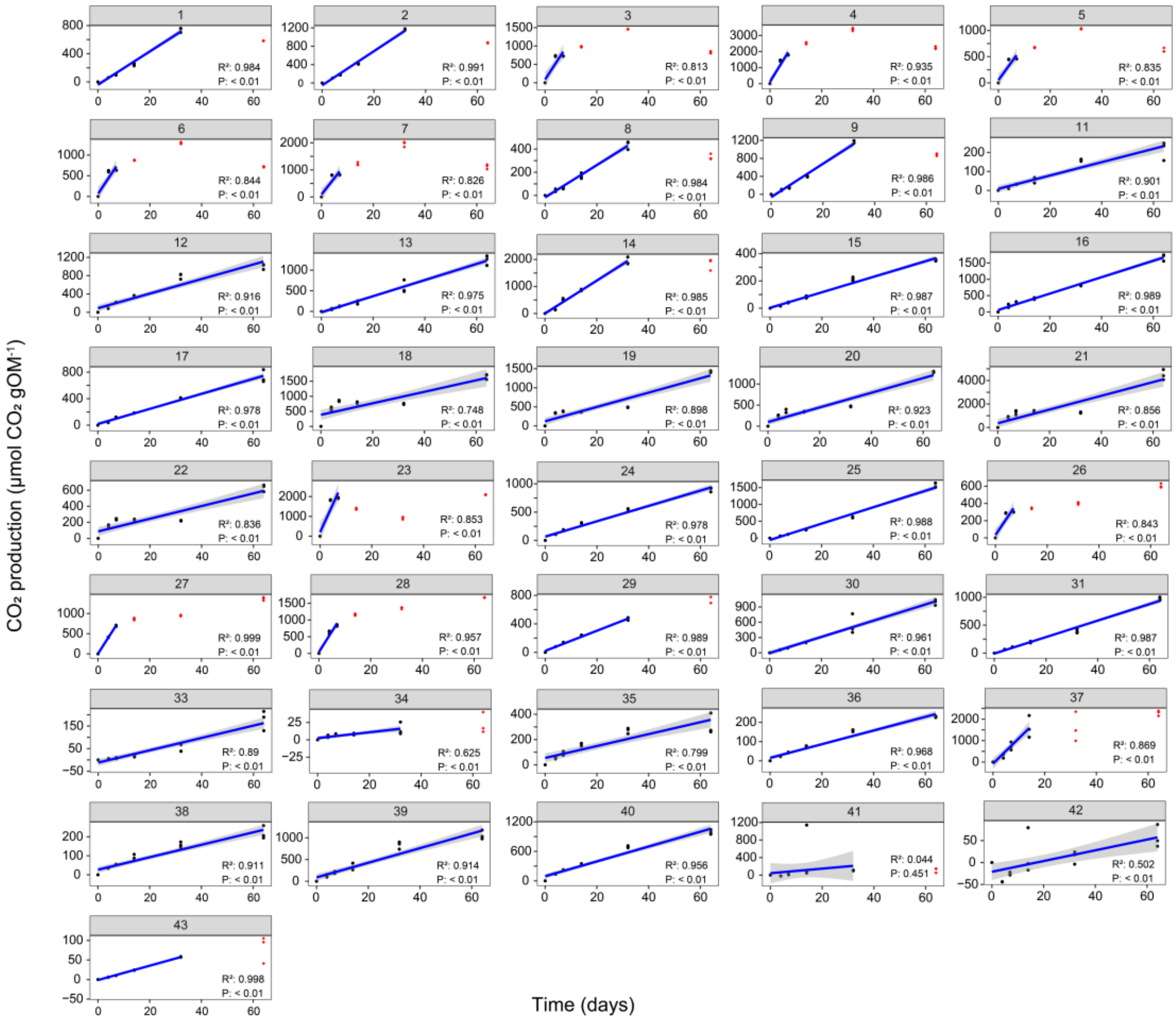

Figure S3. Linear regression of CO_2 production (µmol CO_2 g organic matter^{-1}) over time (days). Black points represent data included in the regression analysis, while red points indicate excluded time points where CO_2 and/or O_2 concentrations deviated from linearity based on segmented regression analysis, suggesting a change in gas dynamics. Each panel corresponds to a different sample, with R^2 and p-values reported for the fitted linear models. Shaded areas around the regression line represent 95% confidence intervals.

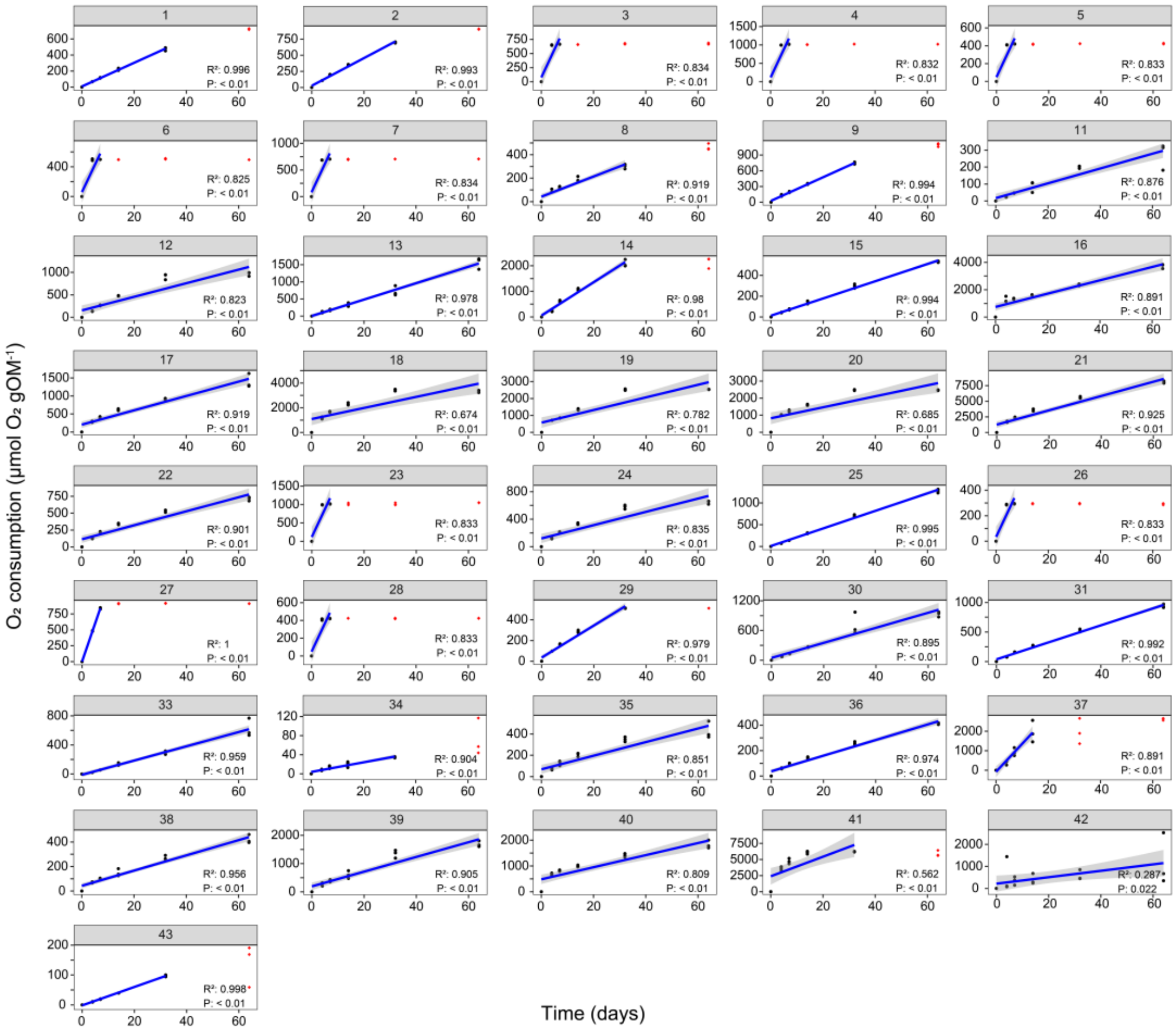

Figure S4. Linear regression of O_2 consumption (µmol O_2 g organic matter^{-1}) over time (days). Black points represent data included in the regression analysis, while red points indicate excluded time points where CO_2 and/or O_2 concentrations deviated from linearity based on segmented regression analysis, suggesting a change in gas dynamics. Each panel corresponds to a different sample, with R^2 and p-values reported for the fitted linear models. Shaded areas around the regression line represent 95% confidence intervals.

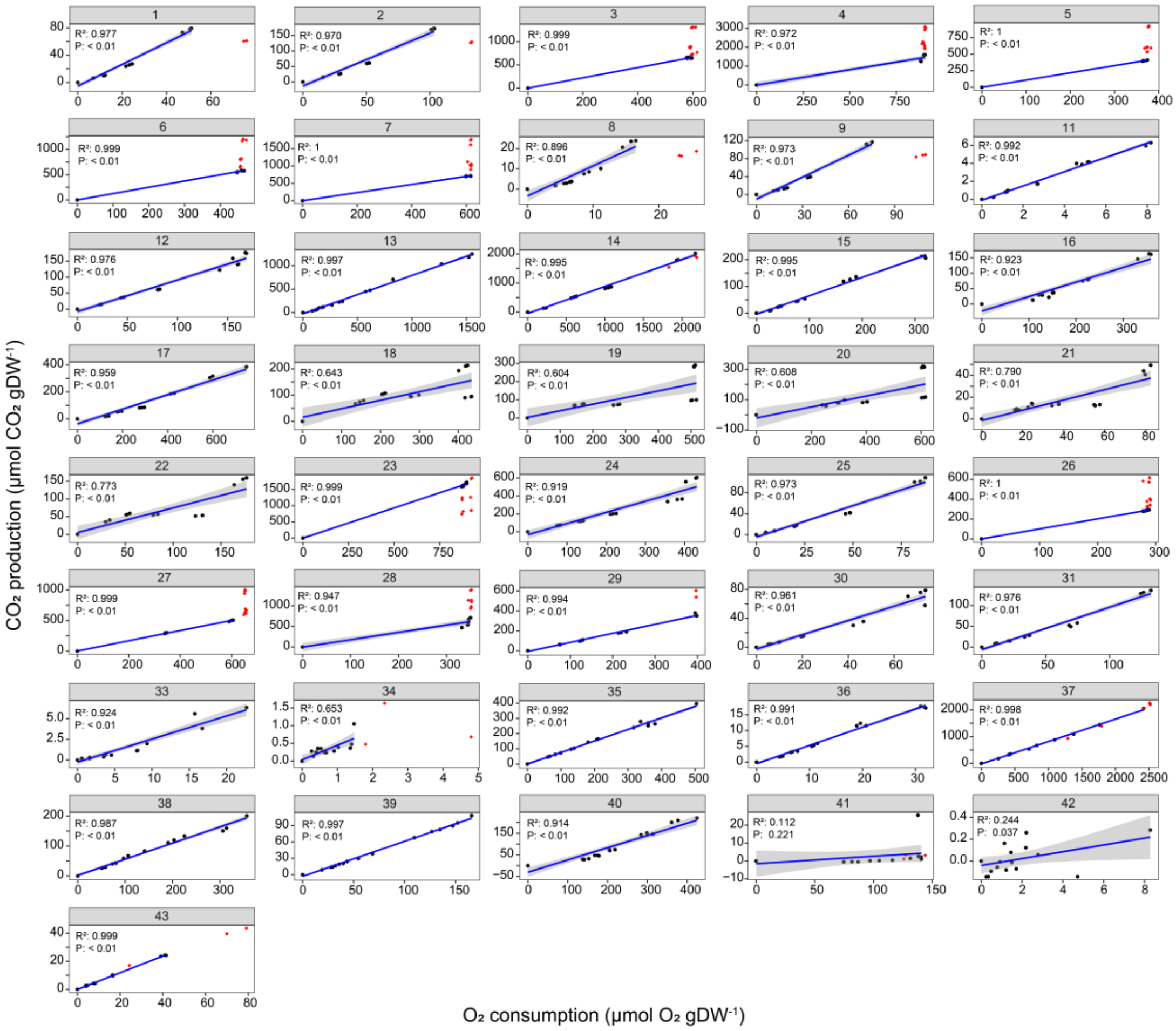

Figure S5. Linear regression of CO_2 production (µmol CO_2 g dry weight^{-1}) over O_2 consumption (µmol O_2 g dry weight^{-1}). Black points represent data included in the regression analysis, while red points indicate excluded time points where CO_2 and/or O_2 concentrations deviated from linearity based on segmented regression analysis, suggesting a change in gas dynamics. Each panel corresponds to a different sample, with R^2 and p-values reported for the fitted linear models. Shaded areas around the regression line represent 95% confidence intervals. The slopes of these regressions were used to calculate the respiratory quotient (RQ) across samples.

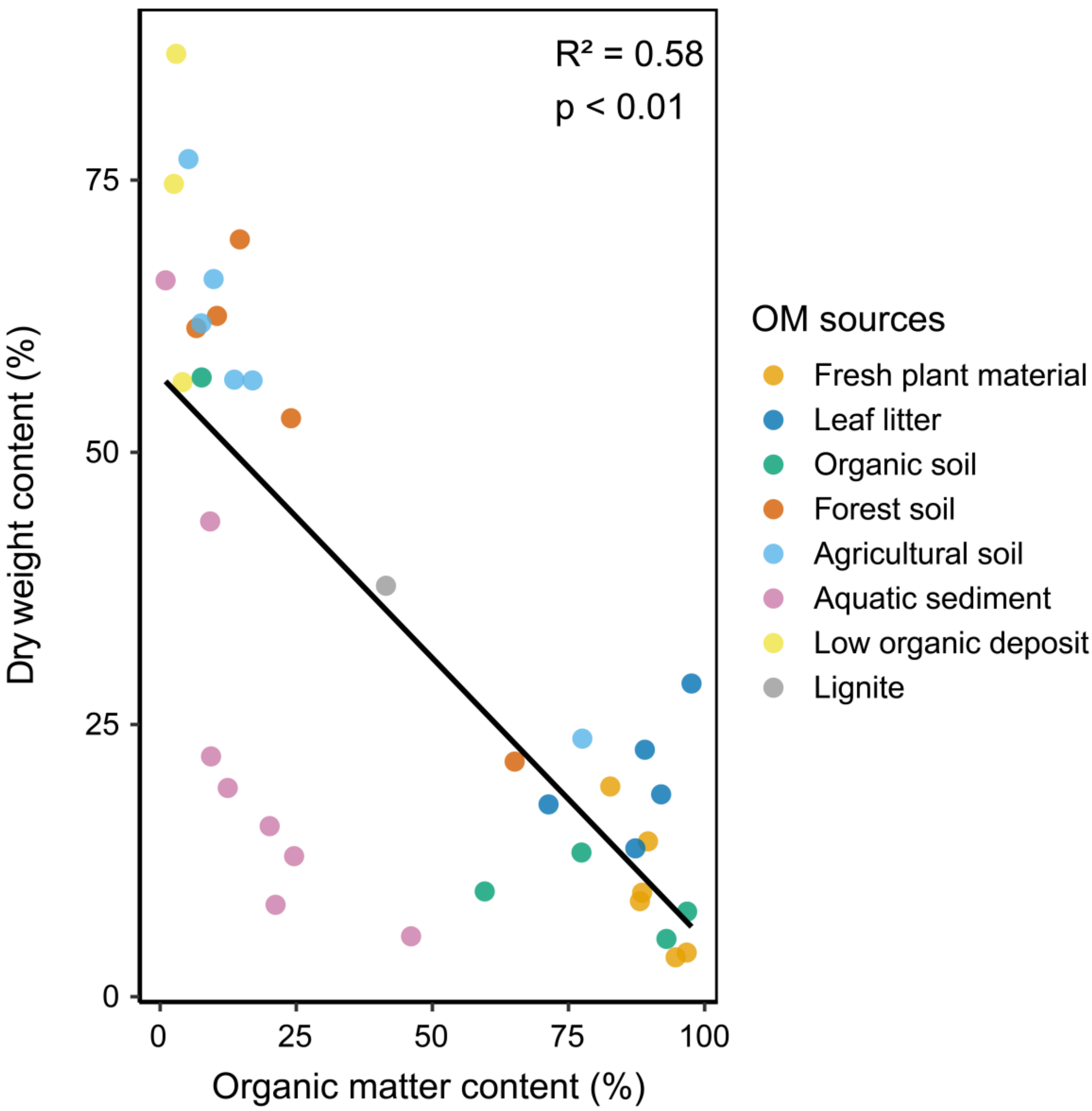

Figure S6. Relationship between organic matter content (%) and dry weight content (%) across different OM sources. Each point represents a sample, colored by OM sources. The black line shows the linear regression fit ($R^2 = 0.58$, $p < 0.01$, n=39), indicating a significant relationship between organic matter content and dry weight.

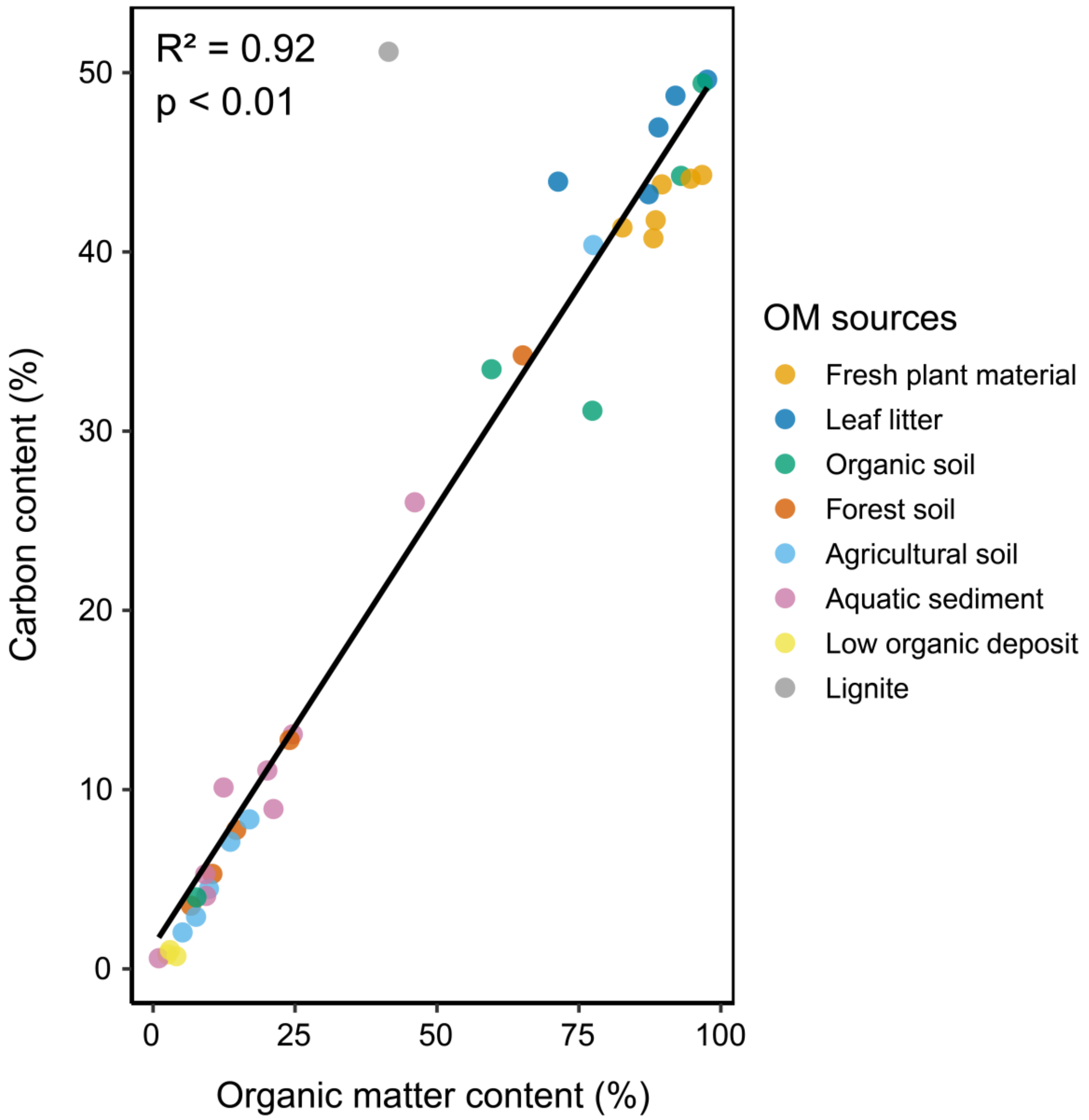

Figure S7. Relationship between organic matter content (%) measured with loss on ignition (LOI) and carbon content (%) measured with Elemental Analyzer across studied materials. Each point represents a sample, color-coded by OM source. The black line indicates the linear regression fit ($R^2 = 0.92$, $p < 0.01$, $n=39$).

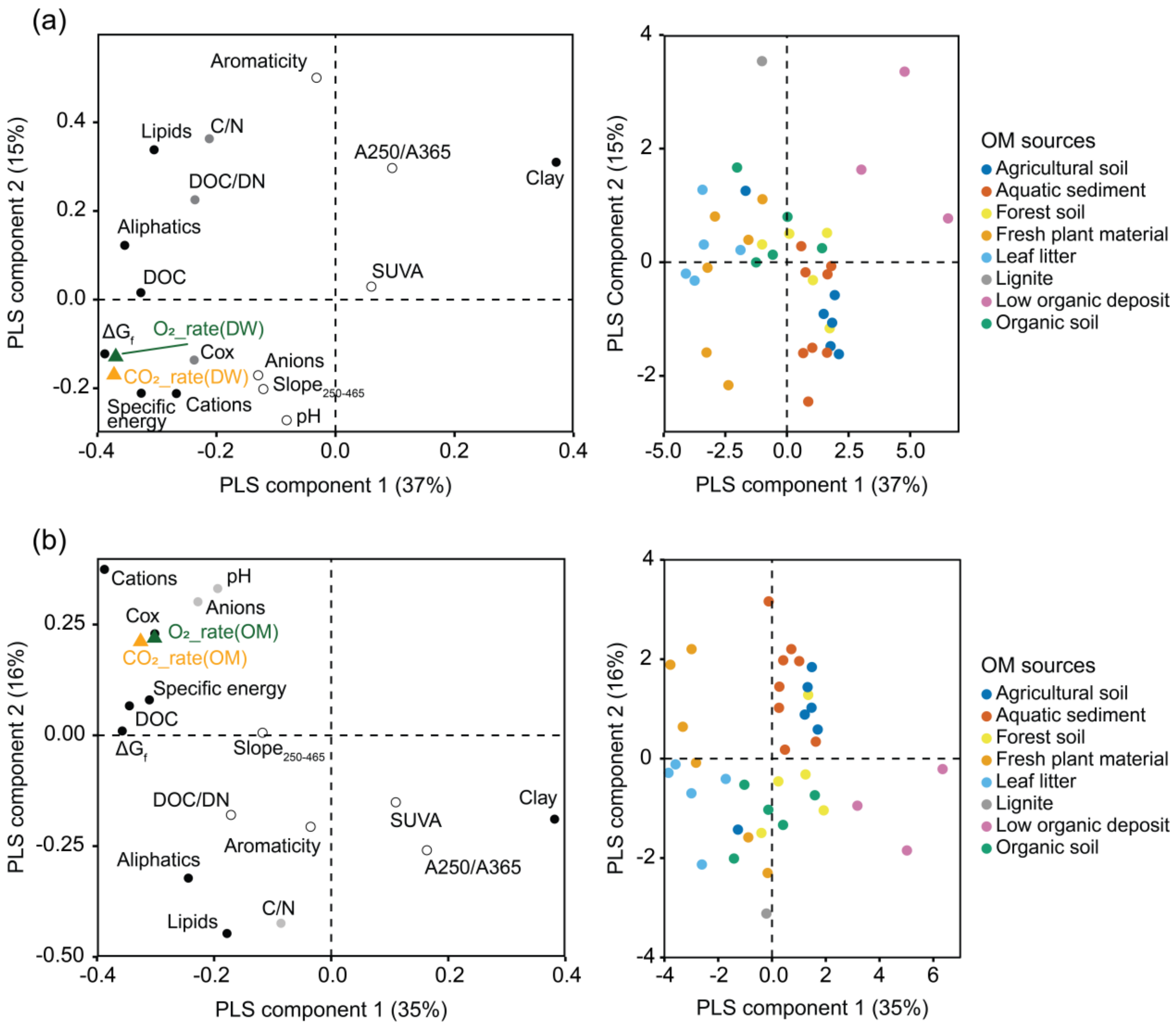

Figure S8. Partial least squares (PLS) model predicting mineralization and respiration rates based on organic matter (OM) bulk properties, FTIR proxies, leachate chemistry, and dissolved organic matter (DOM) characteristics. (a) Mass-specific CO_2 and O_2 rates, denoted as CO_2_rate (orange triangle) and O_2_rate (green triangle). (b) OM-specific CO_2 and O_2 rates, denoted as CO_2_rate (orange triangle) and O_2_rate (green triangle). The left panels show PLS loading plots, where predictor variables are positioned based on their contributions to the first two PLS components (percent variation explained in parentheses). Black circles indicate highly influential variables (VIP ≥ 1), grey circles represent moderately influential variables ($1 > \text{VIP} \geq 0.8$), and white circles represent less influential variables (VIP < 0.8). The right panels display

PLS score plots showing sample distribution along the first two PLS components, color-coded by OM source.

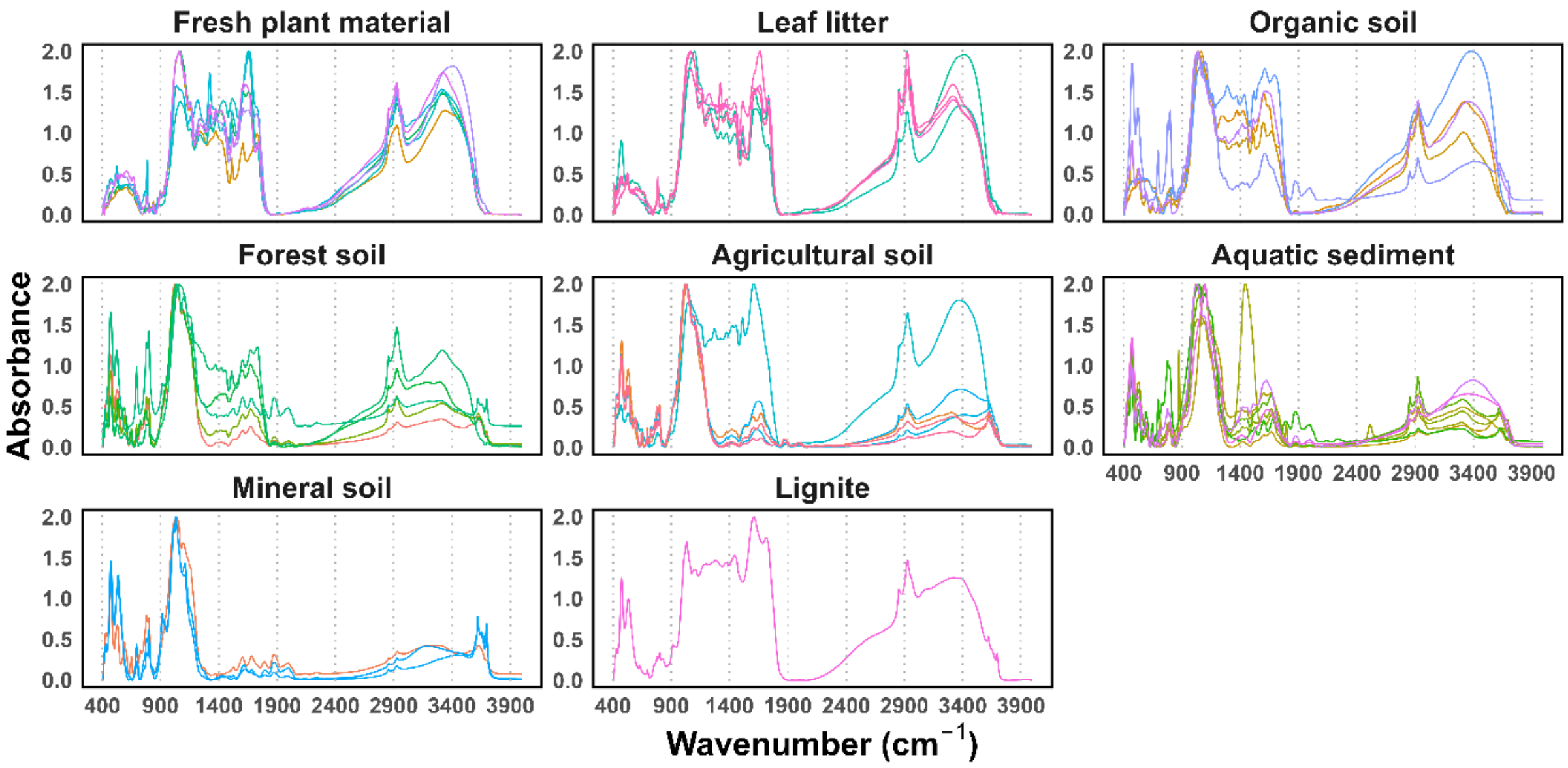

Figure S9. Fourier-transform infrared (FTIR) spectra of environmental samples grouped by OM source. Spectra are shown as absorbance (arbitrary units) across the infrared wavenumber range of 400–4000 cm^{-1}. Each line represents an individual sample within the respective OM source. The spectral profiles reflect differences in molecular vibrations associated with organic and inorganic functional groups present in the samples.

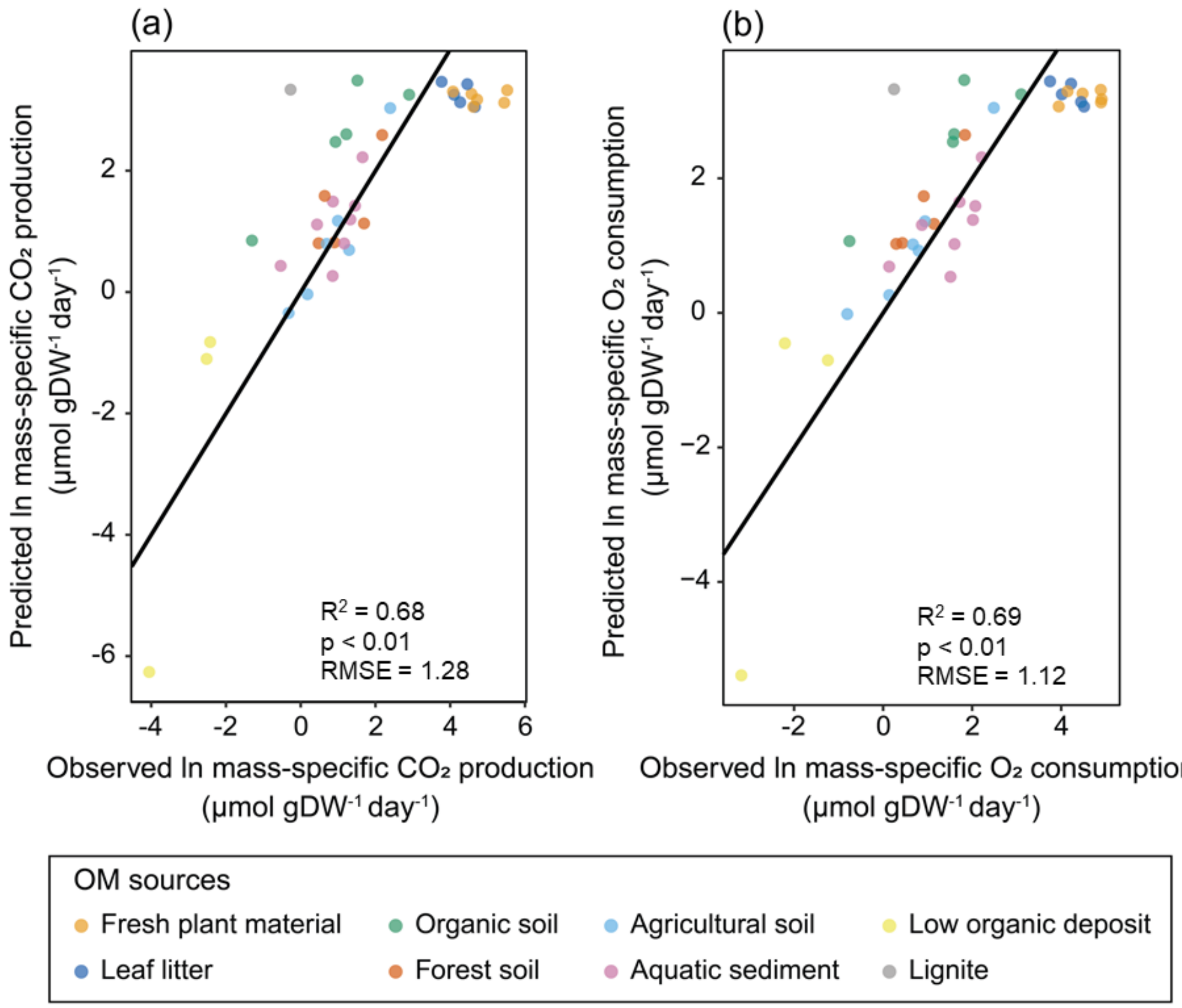

Figure S10. Observed versus predicted mineralization and respiration rates on ln scale across different OM sources as a function of Gibbs free energy of formation (ΔG_f). Panels (a) and (b) show mass-specific CO_2 mineralization and O_2 respiration rates, respectively. Colors represent different OM sources, as indicated in the legend. The solid black line represents the 1:1 relationship, where perfect prediction would align all points. The coefficient of determination (R^2), significance level (*p-value*) and root mean square error (RMSE) in ln-units are displayed in each panel.

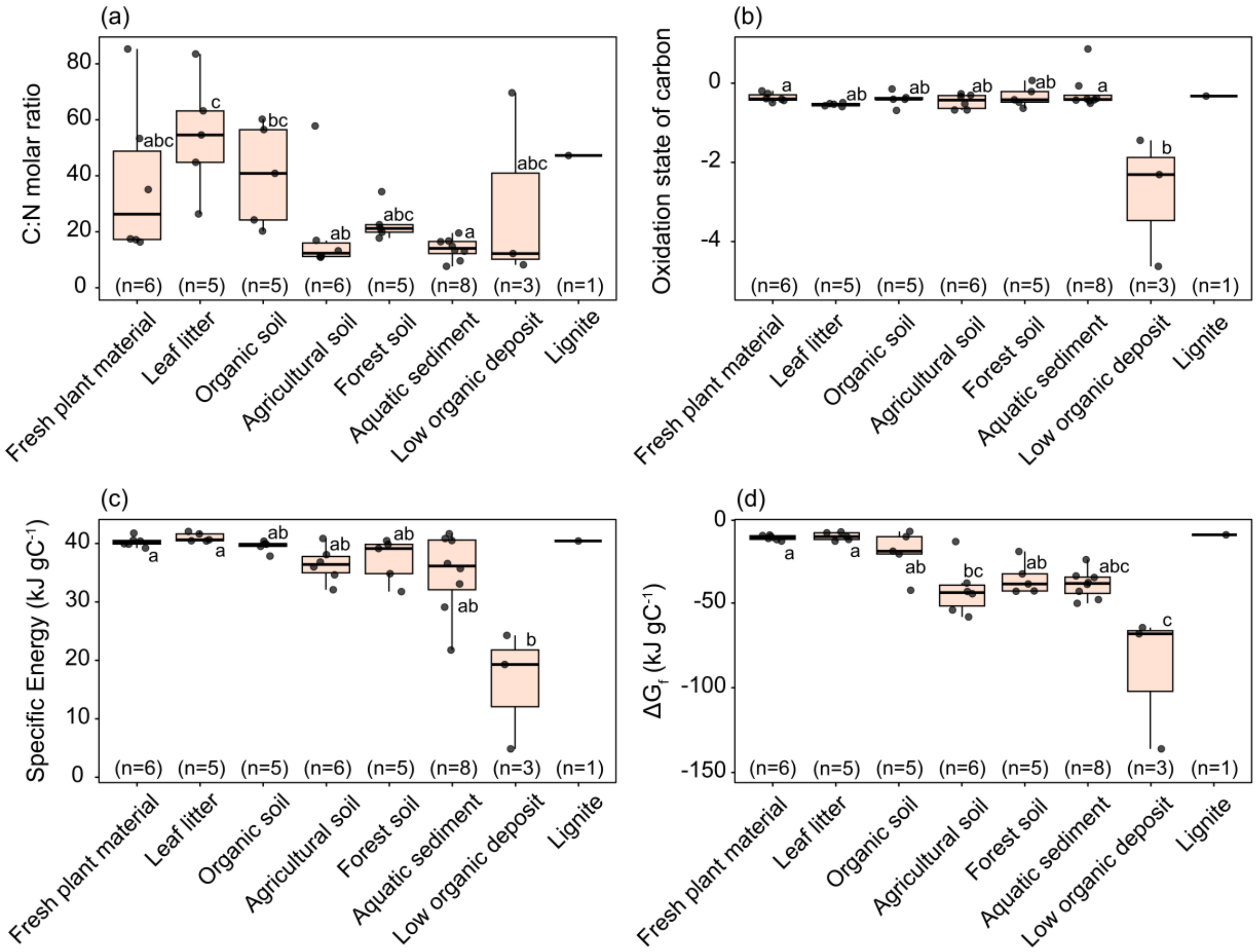

Figure S11. Organic matter properties across different environmental sources. Each boxplot displays the median (central line), interquartile range (box), and whiskers extending to 1.5 times the interquartile range. Points represent individual data values, showing variability within each OM source. Each panel represents a different characteristic related to organic matter bulk composition and energetic properties. (a) Carbon to nitrogen (C:N) molar ratio, (b) oxidation state of carbon (C_{ox}), (c) specific energy content (kJ gC^{-1}) and (d) Gibbs free energy of OM formation (ΔG_f). Letters above boxplots indicate statistical groupings based on Dunn's test following a Kruskal-Wallis analysis, where different letters represent significant differences ($p < 0.05$). Sample sizes (n) for each category are indicated above the x-axis.

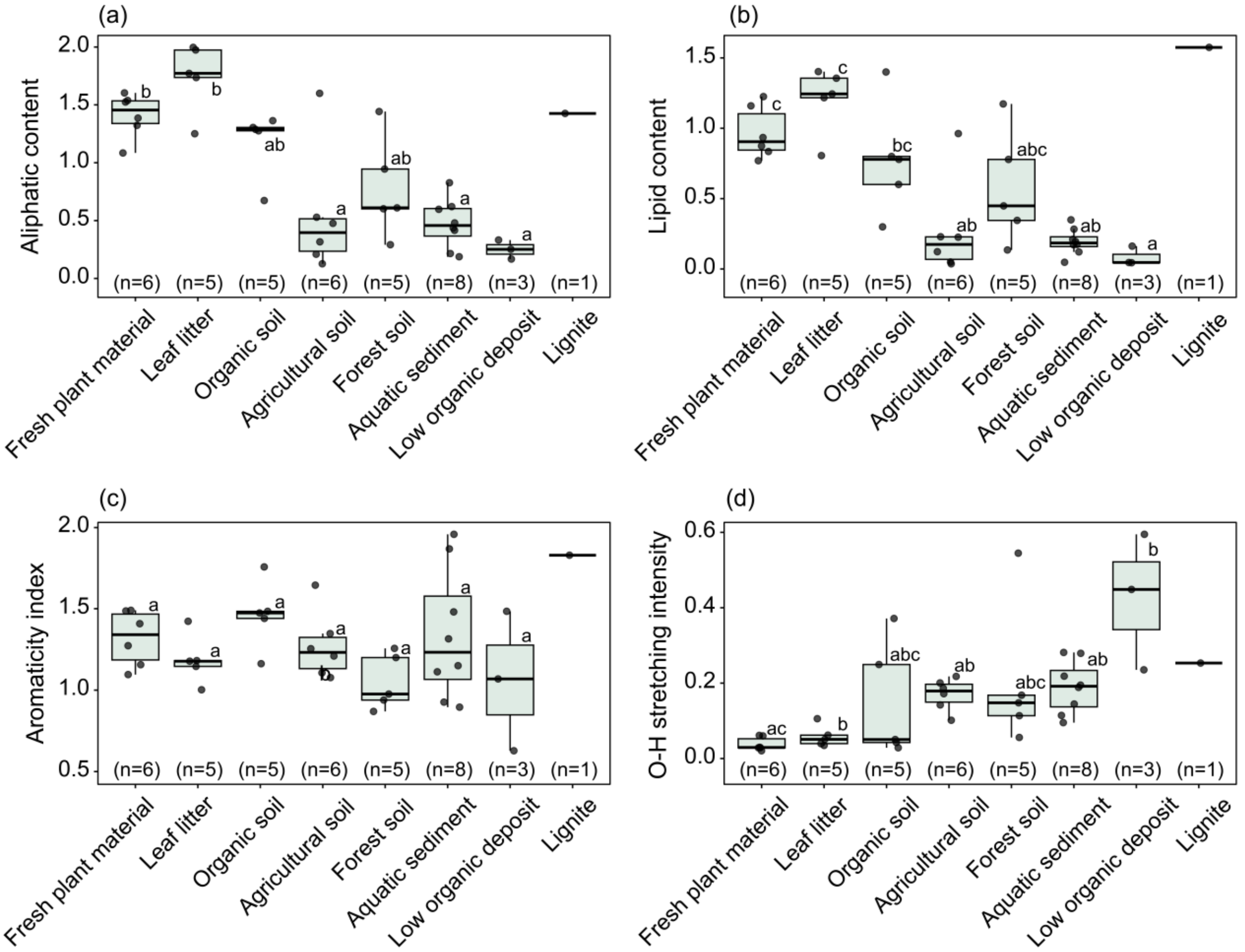

Figure S12. Boxplots showing FTIR-derived organic matter properties across different environmental sources. Each boxplot displays the median (central line), interquartile range (box), and whiskers extending to 1.5 times the interquartile range. Points represent individual data values, showing variability within each OM source. Each panel represents a different chemical proxy obtained from FTIR spectra. (a) Aliphatic content, (b) lipid content, (c) aromaticity index, and (d) O-H stretching intensity in clay minerals. Letters above boxplots indicate statistical groupings based on Dunn's test following a Kruskal-Wallis analysis, where different letters represent significant differences ($p < 0.05$). Sample sizes (n) for each category are indicated above the x-axis.

Table S3. Summary of linear models predicting CO_2 mineralization and O_2 respiration rates (ln-transformed) as a function of Gibbs free energy of formation (ΔG_f). Models (a) and (b) represent mass-specific rates (per gram dry weight). Model performance is indicated by R^2, adjusted R^2, F-statistic, and root mean square error (RMSE). Regression coefficients, standard errors, t-values, and p-values are provided for each model.

Model a: lm(ln(CO_2_DW) ~ ΔG_f)

R^2	0.68			
R^2 adjusted	0.67			
F(1,37)	77.71			
RMSE-ln (unit)	1.28			
	Estimate	Std. error	t value	Pr(>|t|)
Intercept	4.076115	0.345536	11.796	< 0.001
ΔG_f	0.075972	0.008618	8.815	< 0.001

Model b: lm(ln(O_2_DW) ~ ΔG_f)

R^2	0.69			
R^2 adjusted	0.69			
F(1,37)	83.78			
RMSE-ln (unit)	1.12			
	Estimate	Std. error	t value	Pr(>|t|)
Intercept	3.996673	0.302122	13.229	< 0.001
ΔG_f	0.068971	0.007535	9.153	< 0.001

Table S4. Summary of linear models predicting CO_2 mineralization and O_2 respiration rates (ln-transformed) as a function of Gibbs free energy of formation (ΔG_f) excluding the "low organic deposition" sample with the most negative ΔG_f and slowest decomposition rates. Models (a) and (b) represent mass-specific rates (per gram dry weight). Model performance is indicated by R^2, adjusted R^2, F-statistic, and root mean square error (RMSE). Regression coefficients, standard errors, t-values, and p-values are provided for each model.

Model a: lm(ln(CO_2_DW) ~ ΔG_f)

R^2	0.67			
R^2 adjusted	0.66			
F(1,36)	73.29			
RMSE-ln (unit)	1.19			
	Estimate	Std. error	t value	Pr(>|t|)
Intercept	4.59388	0.38132	12.047	< 0.001
ΔG_f	0.09574	0.01118	8.561	< 0.001

Model b: lm(ln(O_2_DW) ~ ΔG_f)

R^2	0.70			
R^2 adjusted	0.69			
F(1,36)	87.05			
RMSE-ln (unit)	1.01			
	Estimate	Std. error	t value	Pr(>|t|)
Intercept	4.513570	0.324180	13.92	< 0.001
ΔG_f	0.088711	0.009508	9.33	< 0.001

Table S5. Summary of linear models predicting CO_2 mineralization and O_2 respiration rates (ln-transformed) as a function of Gibbs free energy of formation (ΔG_f) and the concentration of dissolved organic carbon (DOC). Models (a) and (b) represent mass-specific rates (per gram dry weight). Model performance is indicated by R^2, adjusted R^2, F-statistic, and root mean square error (RMSE) in ln-units. Regression coefficients, standard errors, t-values, p-values, and variance inflation factor (VIF) values are provided for each model.

Model a: lm(ln(CO_2_DW) ~ ΔG_f + DOC)

			VIF (ΔG_f)	VIF (DOC)
R^2	0.77		1.16	1.16
R^2 adjusted	0.76			
F(2,36)	60.44			
RMSE-ln (unit)	4.20			
	Estimate	Std. error	t value	Pr(>|t|)
Intercept	3.355031	0.350592	9.57	< 0.001
ΔG_f	0.064485	0.007959	8.102	< 0.001
DOC	0.00479	0.001254	3.821	< 0.001

Model b: lm(ln(O_2_DW) ~ ΔG_f + DOC)

			VIF (ΔG_f)	VIF (DOC)
R^2	0.77		1.16	1.16
R^2 adjusted	0.76			
F(2,36)	59.78			
RMSE-ln (unit)	3.86			
	Estimate	Std. error	t value	Pr(>|t|)
Intercept	3.416173	0.315867	10.815	< 0.001
ΔG_f	0.059724	0.007171	8.329	< 0.001
DOC	0.003856	0.001129	3.414	< 0.001

References

(1) Korth, B.; Maskow, T.; Günther, S.; Harnisch, F. Estimating the Energy Content of Wastewater Using Combustion Calorimetry and Different Drying Processes. *Front. Energy Res.* **2017**, *5*. https://doi.org/10.3389/fenrg.2017.00023.

(2) Lorenz, M.; Maskow, T.; Thiele-Bruhn, S. Energy Stored in Soil Organic Matter Is Influenced by Litter Quality and the Degree of Transformation – A Combustion Calorimetry Study. *Geoderma* **2024**, *443*, 116846. https://doi.org/10.1016/j.geoderma.2024.116846.

(3) Sander, R. Compilation of Henry's Law Constants (Version 5.0.0) for Water as Solvent. *Atmospheric Chemistry and Physics* **2023**, *23* (19), 10901–12440. https://doi.org/10.5194/acp-23-10901-2023.

(4) Polsenaere, P.; Abril, G. Modelling CO2 Degassing from Small Acidic Rivers Using Water pCO2, DIC and δ13C-DIC Data. *Geochimica et Cosmochimica Acta* **2012**, *91*, 220–239. https://doi.org/10.1016/j.gca.2012.05.030.

(5) Abril, G. Comments on: "Underwater Measurements of Carbon Dioxide Evolution in Marine Plant Communities: A New Method" by J. Silva and R. Santos [Estuarine, Coastal and Shelf Science 78(2008) 827–830]. *Estuarine, Coastal and Shelf Science* **2009**, *82* (2), 357–360. https://doi.org/10.1016/j.ecss.2008.11.027.

(6) Ouisse, V.; Migné, A.; Davoult, D. Comparative Study of Methodologies to Measure *in Situ* the Intertidal Benthic Community Metabolism during Immersion. *Estuarine, Coastal and Shelf Science* **2014**, *136*, 19–25. https://doi.org/10.1016/j.ecss.2013.10.032.

(7) Masiello, C. A.; Gallagher, M. E.; Randerson, J. T.; Deco, R. M.; Chadwick, O. A. Evaluating Two Experimental Approaches for Measuring Ecosystem Carbon Oxidation State and Oxidative Ratio. *Journal of Geophysical Research: Biogeosciences* **2008**, *113* (G3). https://doi.org/10.1029/2007JG000534.

(8) Balk, P. *Removal of Organic Matter by NaOCl: An Improved Method to Characterize Organic Microbial Food Sources*; MSc thesis **2024,** Uppsala University; https://uu.diva-portal.org/smash/record.jsf?pid=diva2%3A1868539&dswid=3126.

(9) Worrall, F.; Moody, C. S.; Clay, G. D.; Burt, T. P.; Kettridge, N.; Rose, R. Thermodynamic Control of the Carbon Budget of a Peatland. *Journal of Geophysical Research: Biogeosciences* **2018**, *123* (6), 1863–1878. https://doi.org/10.1029/2017JG003996.

(10) Informatics, N. O. of D. and. *NIST Chemistry WebBook*. https://webbook.nist.gov/chemistry/ (accessed 2025-06-06).

(11) Lehmann, J.; Solomon, D. Chapter 10 - Organic Carbon Chemistry in Soils Observed by Synchrotron-Based Spectroscopy. In *Developments in Soil Science*; Singh, B., Gräfe, M., Eds.; Synchrotron-Based Techniques in Soils and Sediments; Elsevier, 2010; Vol. 34, pp 289–312. https://doi.org/10.1016/S0166-2481(10)34010-4.

(12) Margenot, A. J.; Parikh, S. J.; Calderón, F. J. Fourier-Transform Infrared Spectroscopy for Soil Organic Matter Analysis. *Soil Science Society of America Journal* **2023**, *87* (6), 1503–1528. https://doi.org/10.1002/saj2.20583.

(13) Samuel Obeng, A.; Dunne, J.; Giltrap, M.; Tian, F. Soil Organic Matter Carbon Chemistry Signatures, Hydrophobicity and Humification Index Following Land Use Change in Temperate Peat Soils. *Heliyon* **2023**, *9* (9), e19347. https://doi.org/10.1016/j.heliyon.2023.e19347.

(14) Weishaar, J. L.; Aiken, G. R.; Bergamaschi, B. A.; Fram, M. S.; Fujii, R.; Mopper, K. Evaluation of Specific Ultraviolet Absorbance as an Indicator of the Chemical Composition and Reactivity of Dissolved Organic Carbon. *Environ. Sci. Technol.* **2003**, *37* (20), 4702–4708. https://doi.org/10.1021/es030360x.

(15) Peuravuori, J.; Pihlaja, K. Molecular Size Distribution and Spectroscopic Properties of Aquatic Humic Substances. *Analytica Chimica Acta* **1997**, *337* (2), 133–149. https://doi.org/10.1016/S0003-2670(96)00412-6.

(16) Helms, J. R.; Stubbins, A.; Ritchie, J. D.; Minor, E. C.; Kieber, D. J.; Mopper, K. Absorption Spectral Slopes and Slope Ratios as Indicators of Molecular Weight, Source, and Photobleaching of Chromophoric Dissolved Organic Matter. *Limnology and Oceanography* **2008**, *53* (3), 955–969. https://doi.org/10.4319/lo.2008.53.3.0955.
